# Reprogramming of the wheat transcriptome in response to infection with *Claviceps purpurea*, the causal agent of ergot

**DOI:** 10.1186/s12870-021-03086-3

**Published:** 2021-07-02

**Authors:** Eleni Tente, Nelzo Ereful, Anyela Camargo Rodriguez, Paul Grant, Donal M. O’Sullivan, Lesley A. Boyd, Anna Gordon

**Affiliations:** 1 NIAB, 93 Lawrence Weaver Road, Cambridge, CB3 0LE UK; 2 Department of Plant Sciences, University of Cambridge, Downing Street, Cambridge, CB2 3EA UK; 3 Philippine Genome Center, Plant Physiology Laboratory, Institute of Plant Breeding, University of the Philippines, Los Baños, Laguna The Philippines; 4 Present Address: Microsoft Research, 21 Station Road, Cambridge, CB1 2FB UK; 5 School of Agriculture, Policy and Development, University of Reading, Whiteknights, Reading, RG6 6AR UK

**Keywords:** *Claviceps purpurea*, Ergot, Fungal pathogen, *Triticum aestivum*, Wheat

## Abstract

**Background:**

Ergot, caused by the fungal pathogen *Claviceps purpurea*, infects the female flowers of a range of cereal crops, including wheat. To understand the interaction between *C. purpurea* and hexaploid wheat we undertook an extensive examination of the reprogramming of the wheat transcriptome in response to *C. purpurea* infection through floral tissues (i.e. the stigma, transmitting and base ovule tissues of the ovary) and over time.

**Results:**

*C. purpurea* hyphae were observed to have grown into and down the stigma at 24 h (H) after inoculation. By 48H hyphae had grown through the transmitting tissue into the base, while by 72H hyphae had surrounded the ovule. By 5 days (D) the ovule had been replaced by fungal tissue. Differential gene expression was first observed at 1H in the stigma tissue. Many of the wheat genes differentially transcribed in response to *C. purpurea* infection were associated with plant hormones and included the ethylene (ET), auxin, cytokinin, gibberellic acid (GA), salicylic acid and jasmonic acid (JA) biosynthetic and signaling pathways. Hormone-associated genes were first detected in the stigma and base tissues at 24H, but not in the transmitting tissue. Genes associated with GA and JA pathways were seen in the stigma at 24H, while JA and ET-associated genes were identified in the base at 24H. In addition, several defence-related genes were differential expressed in response to *C. purpurea* infection, including antifungal proteins, endocytosis/exocytosis-related proteins, NBS-LRR class proteins, genes involved in programmed cell death, receptor protein kinases and transcription factors. Of particular interest was the identification of differential expression of wheat genes in the base tissue well before the appearance of fungal hyphae, suggesting that a mobile signal, either pathogen or plant-derived, is delivered to the base prior to colonisation.

**Conclusions:**

Multiple host hormone biosynthesis and signalling pathways were significantly perturbed from an early stage in the wheat – *C. purpurea* interaction. Differential gene expression at the base of the ovary, ahead of arrival of the pathogen, indicated the potential presence of a long-distance signal modifying host gene expression.

**Supplementary Information:**

The online version contains supplementary material available at 10.1186/s12870-021-03086-3.

## Background

Ergot, caused by the fungal pathogen *Claviceps purpurea*, is an ear disease of grasses and cereal, and infects a number of economically important cereal crops, including wheat, barley and rye [1, 2]. Ergot can lead to significant economic loss, grain being rejected due to contamination with ergot sclerotia, the over-wintering fungal structure [3]. While sclerotia can generally be removed from grain by standard cleaning methods: colour sorting and gravity tables [4–6], sclerotia of a similar size to the seed are difficult to separate. Sclerotia contain a range of ergot alkaloids that are highly toxic to humans and animals [4, 7]. These alkaloids are responsible for the condition ergotism, which during the Middle Ages was known as St Anthony’s Fire. Symptoms of ergotism include gangrenous extremities, convulsions, psychosis and eventually death. Outbreaks were especially prevalent in the Middle Ages due to a diet high in rye [8]. In addition, recent findings suggest that ergot alkaloids, produced by the fungus and found at high concentrations in sclerotia, can find their way onto otherwise “healthy” grain [9].

**Fig. 1:**
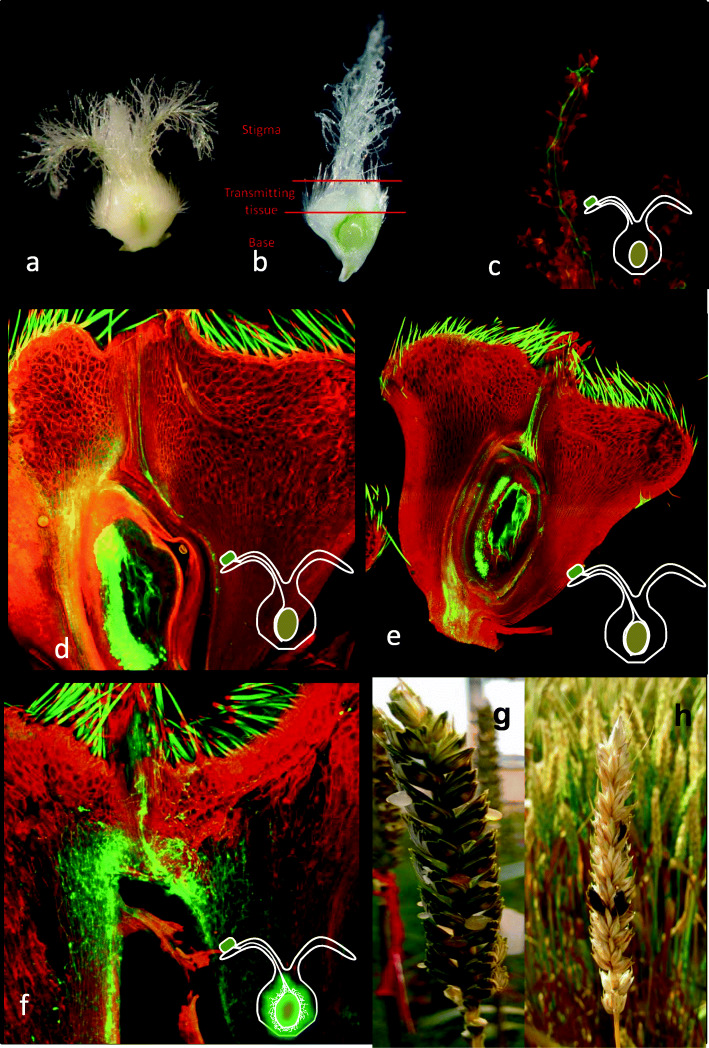
*Claviceps purpurea* infection of wheat. **a** Wheat ovary. **b** Longitudinal section of ovary showing stigma, transmitting and base tissue. Confocal images of wheat infected with *C. purpurea* at **(c)** 24 h, **(d)** 48 h, **(e)** 72 h and **(f)** 5 days after inoculation. Images stained with propidium iodide and aniline blue. At 24 h *C. purpurea* conidia have germinated and a germ tube grown down the stigma hairs **(c)**. By 48 h hyphae had grown through the transmitting tissue and entered the base tissue **(d)**, while at 72 h the ovule is surrounded by fungal hyphae **(e)**. By 5 days after inoculation the ovule has been completely replaced by fungal tissue **(f)**. **g** Wheat ear extruding honeydew. **h** Wheat ear with sclerotia

*C. purpurea* gains entry during anthesis, infecting the flower’s female tissues and replacing the seed with an ergot sclerotia [1]. Cereals such as rye, that exhibit open flowering, are therefore particularly at risk of infection, as are hybrid cereal seed production systems, such as those developed for barley and wheat [10]. *C. purpurea* is believed to exhibit a biotrophic lifestyle, keeping the floral tissues alive while it draws nutrients from the plant, and does not induce host tissue necrosis [1]. Spores germinate on stigma, penetrate stigmatic hairs and grow down the style to the transmitting tissue of the ovary (Fig. 1). *C. purpurea* grows mainly intercellularly, although invasive hyphae, which are completely enclosed by the host plasma membrane, have been documented [11]. Within three days of spores landing on the stigma hyphae have completely overwhelmed the ovary and begin to branch. Between 5 and 7 days post-infection the fungus enters the sphacelial stage, manifesting itself as a soft white tissue that begins producing asexual conidiospores. The conidiospores are exuded from florets in a sugary liquid called honeydew [1]. The honeydew enables *C. purpurea* to disperse conidiospores to other receptive flowers, most likely with the help of insect vectors and rain splash [12, 13]. After approximately 2 weeks a hardened, dark sclerotium is formed where a seed would have developed. These sclerotia, also known as ergots, produce the sexual reproduction structures that give rise to ascospores [12].

The interaction between host plant and invading pathogen involves continuous, two-way communication. Initial plant recognition of pathogen-associated molecular patterns (PAMPs) triggers PAMP-Triggered Immunity (PTI). A pathogen can suppress and/or avoid PTI through the delivery of pathogen effectors (Effector Triggered Suppression – ETS). Effectors are thought to suppress PTI either by preventing detection of the PAMPs by the host [14], or by affecting downstream PTI-signaling pathways [15]. However, it is becoming increasingly apparent that the role of effectors goes beyond suppression of plant defence, having a strategic role in modifying the plant environment to create conditions conducive for pathogen growth and reproduction. Therefore, many of the plant genes induced upon pathogen infection, once thought to be required for defence, are actually required for infection by the pathogen [15].

As a second line of defence plants have evolved an effector recognition system, mediated by host resistance (R-) genes that recognise effectors either directly or indirectly, leading to Effector Triggered Immunity (ETI) [16]. Downstream responses often associated with PTI and ETI include a rapid influx of calcium ions, a burst of ROS [17], deposition of callosic cell wall appositions at sites of attempted pathogen infection, as well as activation of a MAPK signaling cascade that triggers expression of WRKY-type transcription factors, key regulators of plant defence [18]. WRKY transcription factors elicit defence responses such as the generation of nitric oxide, production of antimicrobial compounds, and the hypersensitive or programmed cell death response [18].

Plants have also established complex phytohormone-regulated signaling pathways to control defence responses [15]. In return, the pathogen has developed strategies to manipulate phytohormone-regulated defense, delivering effectors that allow the pathogen to evade, hijack or disrupt hormone signaling pathways [15]. The plant hormones salicylic acid (SA), jasmonic acid (JA) and ethylene (ET) have well established roles in the regulation of plant defence. SA is known for its role in the activation of defence responses against biotrophic pathogens and for the establishment of systemic acquired resistance (SAR) [19]. JA and ET primarily activate defence responses against necrotrophic pathogens and herbivorous insects, and have been found to act in a mutually antagonistic manner with SA [20]. Auxins and Giberellic Acid (GA) have also been shown to play a role in plant defence, with the overexpression of the auxin conjugating protein GH3–8 in rice leading to enhanced resistance to bacterial blight disease [21].

In *Arabidopsis* resistance to biotrophs and susceptibility to necrotrophs was regulated by a shift in the balance between JA and SA signaling, which in turn was dependent on GA-dependent degradation of the DELLA proteins [22]. It has been suggested that DELLA proteins are able to bind to the JA suppressor JAZ1, preventing it from interacting with MYC2, a key transcriptional activator of JA responses, thereby leading to the activation of JA-responsive target genes [23]. As the degradation of DELLA proteins is GA-dependent, GA was implemented in this control of JA-responsive target genes. It was also found that the GA insensitive mutant *gid1,* which hyper-accumulates endogenous GA, displays enhanced susceptibility to rice blast [24], while rice plants compromised in GA biosynthesis (i.e. hypo-accumulation of GA) were found to exhibit increased resistance to *M. oryzae* [25].

RNA sequencing (RNASeq) has been successfully used to profile changes in the wheat transcriptome in response to a number of pathogens, including *Zymoseptoria tritici* [26, 27], *Fusarium graminearum* [28], *Puccinia striiformis* and *Blumeria graminis* [29]. While the recent release of an annotated, hexaploid wheat reference genome sequence, RefSeq [30] means that resources are now available to support a detailed and global examination of changes in wheat gene expression in response to pathogen infection.

The aim of this study was to determine the molecular genetic changes that occur in wheat female flowers as *C. purpurea* infection progresses through the tissues of the ovary. The female flowers of a male sterile wheat line were inoculated with an aggressive strain of *C. purpurea*. Female flowers were microscopically examined at specific time points after *C. purpurea* inoculation to follow the infection process through the stigma, the ovary transmitting tissue, to the ovule base. Tissue samples were collected at the same times points from stigma, transmitting and base tissues for RNASeq and differential gene expression analyses. Changes in wheat gene expression were compared across floral tissues and time points, relative to the stages of *C. purpurea* development.

## Results

### Microscopic examination of *Claviceps purpurea* infection of wheat

The percentage of ovaries with *C. purpurea* hyphae in stigma, transmitting and base tissues were scored across time points (Table 1). At 10 mins after inoculation conidia of *C. purpurea* were visible on the stigma, but no hyphal growth was observed. Conidia were observed to have germinated, with hyphae growing into and down the stigma at 24H (Fig. 1c). By 48H hyphae had grown through the transmitting tissue and had entered the base of the ovary (Fig. 1d). By 72H hyphae had surrounded the ovule and occupied much of the base close to the boundary with the rachis, where the vasculature enters the ovary (Fig. 1e). At 5D fungal mycelium has ramified through-out the ovule tissue (Fig. 1f).

**Table 1:** The development of *Claviceps purpurea* infection in female floral tissues over time

Time after *Cp* inoculation	% of ovaries with hyphae visible in stigma tissue	% of ovaries with hyphae visible in transmitting tissue	% of ovaries with hyphae visible in base tissue
10 min (*n* = 12)	0%	0%	0%
1H (*n* = 13)	7.7%	0%	0%
24H (*n* = 2)^a^	100.0%	0%	0%
48H (*n* = 41)	59%	59%	51%
72H (*n* = 57)	87%	87%	87%
5D (*n* = 60)	100%	100%	100%
7D (n = 60)	100%	100%	100%

*n* number of ovaries observed, *H* hours after inoculation, *D* days after inoculation; ^a^only 2 ovary samples were available for the 24H time point

### Quality check of RNAseq libraries

To determine the response of wheat to infection with *C. purpurea* we undertook an RNASeq analysis of female floral tissues – stigma, transmitting and base tissues, at specific time points after *Cp*-inoculation, up until 7D (Table 2). Each tissue by time point interaction was represented by a minimum of two biological replicate RNA libraries. Libraries with an average read coverage of less than 5× were removed from the study. Therefore, the 5H *Cp*- and Mock-inoculated samples were removed from subsequent analyses. The average read coverage of the remaining libraries was 9×, the highest being 29×. Pearson’s coefficient of correlations, using the normalized read counts, were used to compare replicate libraries of each tissue and time point. In general, correlations of 0.90–0.99 were found between replicate libraries. The Mock-inoculated transmitting tissue at 24H had the lowest correlations of 0.80 to 0.83.

**Table 2:** Female floral tissues and time points sampled after *Claviceps purpurea* inoculation

Time points	Mock-inoculated	*Claviceps purpurea* inoculated
T_10 minutes_	Stigma (2)	Stigma (3)
	TT (3)	TT (2)
	Base (3)	Base (3)
T_1 H_	Stigma (3)	Stigma (2)
	TT (3)	TT (3)
	Base (1)	Base (3)
T_5H_	Stigma (2)	Stigma (3)
	TT (2)	TT (3)
	Base (2)	Base (3)
^a^T_24H_	Stigma (3)	Stigma (2)
	TT (3)	TT (3)
	Base (3)	Base (3)
T_48H_	TT (3)	TT (3)
	Base (3)	Base (3)
T_72H_	TT (3)	TT (3)
	Base (3)	Base (3)
T_5D_	TT (2)	TT (3)
	Base (2)	Base (3)
T_7D_	TT (2)	TT (3)
	Base (3)	Base (3)

TT - Transmitting ovary tissue, Base - Ovary tissue. Numbers in brackets are the numbers of replicate samples made into RNA libraries for RNASeq analysis. ^a^Stigma tissues could only be sampled up to 24H, as after 24 h stigma began to collapse. *H* hours after inoculation, *D* days after inoculation

MA plots with Loess curves were generated to determine whether the normalization procedure was adequate with respect to the library size (Additional file 1: Fig. S1; Fig. S2). Samples at the early time points gave symmetrical MA plots with “centered” Loess curves, indicating that the normalization procedure was adequate. However, in the 5D and 7D samples we found bimodal distribution of points in the MA plots due to the presence of RNA transcripts from two biological organisms, wheat and *C. purpurea*. The apparent asymmetry in the MA plots is due to the contrasting transcriptional activities of wheat and *C. purpurea* at these later time points, *C. purpurea* genes being expressed at higher levels as the wheat ovary is replaced by fungal hyphae.

### Establishment of a reference transcriptome for wheat and *Claviceps purpurea*

To check whether there was reciprocal mapping of reads between the wheat and *C. purpurea* transcriptomes we calculated the percentage of wheat reads mapping to the *C. purpurea* reference transcriptome and vice-versa. Only 0.0016% of the wheat reads mapped to the *C. purpurea* transcriptome reference, while 0.037% of the *C. purpurea* reads (from libraires made from ungerminated *C. purpurea* conidia and *C. purpurea* mycelium grown in vitro) aligned to the wheat transcriptome reference. These percentages demonstrate that there is a negligible number of reads cross-mapping between the reference sequences of these two species. The bread wheat variety Chinese Spring IWGSC RefSeq v1 and *C. purpurea* cDNA (Ensembl release 35) transcriptomes were therefore merged to create a single reference transcriptome that was used in the subsequent gene differential expression analysis.

Using this reference transcriptome 95 of the 114 libraries (83%) had read percentage alignment rates from 70 to 85%, particularly at the early timepoints. However, with libraries from 5D and 7D the alignment rates fell to values as low as 36% (Additional file 1: Table S1). The low read alignments were found to be due to a high percentage of *C. purpurea* reads present at these later time points, and a significant number of the *C. purpurea* transcripts not being represented in the *C. purpurea* cDNA reference transcriptome (Ensembl release 35). Reads from the ungerminated *C. purpurea* conidia and *C. purpurea* hyphae grown on artificial media libraries were mapped to the *C. purpurea* cDNA (Ensembl release 35) transcriptomes. Unmapped reads from the two *C. purpurea* libraries were extracted, pooled and de novo assembled to provide a new *Cp*-reference transcriptome. Alignment of the 5D and 7D *Cp*-inoculated libraries to this new wheat-*C. purpurea* transcriptome reference now gave percentage alignments in the range of 85–90%, a big improvement from the original 36–39%. This indicates that a large percentage of the unmapped reads at the latter time-points are derived from *C. purpurea* transcripts that are not represented in the *C. purpurea* transcriptome assembly (Ensembl release 35).

### Spatio-temporal patterns of host, wheat gene expression in response to *Claviceps purpurea* infection

To understand the changes that occur in wheat female flowers upon infection with *C. purpurea,* we undertook a time course experiment to quantify changes in the wheat transcriptome in stigma, transmitting and base tissues of ovaries at 10 min, 1, 24, 48 and 72H, and 5 and 7D after inoculation with a single isolate of *C. purpurea* (Table 2). A pairwise, cross-conditional differential expression analysis was performed, comparing *Cp*- to Mock-inoculated samples in each tissue and at each time point. All differentially expressed genes (DEG) can be found in Additional files 2, 3 and 4.

Wheat genes were observed to be differentially expressed in the three ovary tissues across the 7 day period of *C. purpurea* infection (Additional files 2, 3 and 4). Annotation of these DEG indicated enrichment for a number of functional categories. Prominent among these were classical defence-related genes and wheat genes associated with hormone pathways. Other functional categories included genes associated with photosynthesis, genes involved in oxidation/reduction processes and genes involved in protein phosphorylation (Additional files 2, 3 and 4).

No significant changes in the wheat transcriptome were seen at 10 min after inoculation with *C. purpurea*. At 1H, seven DEG were detected in the stigma, but no DEG were found in the transmitting or base tissues at this time point. Of the seven stigma DEG one was up-regulated, being annotated as a chlorophyll a-b binding protein. Chlorophyll a-b binding protein forms part of the plant’s light harvesting complex, located in the chloroplast, which captures and delivers excitation energy to photosystems I and II (Additional files 2, 3 and 4). However, it is unclear why this gene should be up-regulated in stigma.

Two of the six genes down-regulated in the stigma at 1H were DNA binding transcription factors (TFs) (Additional files 2, 3 and 4). The generic annotation of these TFs makes it difficult to identify the pathways in which they operate, and therefore their potential downstream targets, but they could either result from *Cp* immune-suppressive activity or a host defence response. Also, down-regulated in the stigma at 1H were a myosin protein, known for its role in cytoplasmic streaming [31], a Kelch-like protein, a DnaJ protein and a sucrose synthase. Kelch proteins contain repeat motifs forming β-propeller domains that mediate protein-protein interactions and are involved in a wide array of cellular activities [32]. DnaJ proteins, otherwise known as HSP40s (heat-shock protein 40), are a family of conserved co-chaperones for HSP70s and are known to play diverse roles in stress responses and developmental processes such as flowering [33]. Sucrose synthase has a role in the rapid mobilisation of carbohydrates during defence [34], so may indicate an early attempt by *C. purpurea* to alter the carbohydrate profiles within the floral tissues in support of fungal growth.

**Fig. 2:**
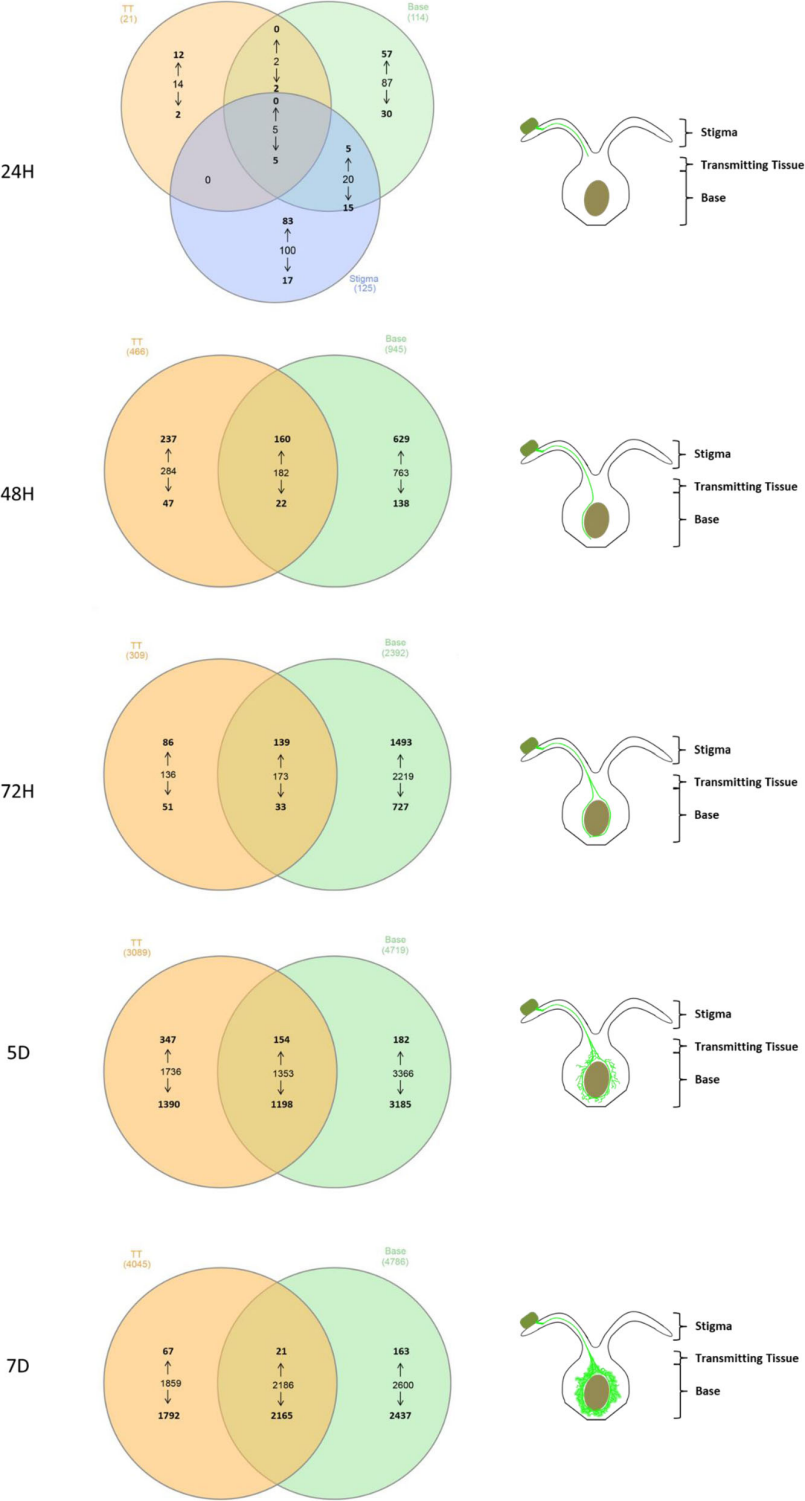
Venn diagram showing the numbers of wheat differentially expressed genes within stigma, transmitting and base ovary tissues at 24H, 48H, 72H, 5D and 7D after inoculation with a single isolate of *Claviceps purpurea*. The arrows pointing up and down designate the numbers of genes that are up- or down-regulated respectively. A schematic representation of the stage of fungal development in the wheat ovary at each time point is shown to the right of each Venn diagram. H = hours; D = days

At subsequent time points differences in the numbers of wheat genes differentially expressed were observed between the ovary tissues, especially at the early time points (Fig. 2). At 24H more genes were differentially expressed in the stigma (125 DEG; 100 genes specific to stigma) and base (114 DEG; 87 genes specific to base) tissues, while few differentially expressed wheat genes were detected in the transmitting tissue (21 DEG; 14 genes specific to transmitting tissue). At 24H *C. purpuea* was observed to have grown into the stigma, however no *C. purpurea* hyphal growth was ever observed in the base tissue at this early time point (Fig. 1). Therefore, changes in wheat gene expression at the base of the ovary, prior to the arrival of fungus, would suggest that a potential mobile signal, either pathogen or plant-derived, is delivered to the base tissue prior to its colonisation by the fungus.

Five DEG were found in common between the stigma, transmitting and base tissues, all being down-regulated (24H; Fig. 2). These included a glycosyl hydrolase (xylanase), a F-box family protein, a myosin and a vesicle-associated membrane protein, all of which can be linked to plant defence responses, with down-regulation fitting with an early suppression of plant defence by *C. purpurea* (Additional files 2, 3 and 4).

Of the 20 DEG in common between the stigma and base tissues at 24H, 5 were up-regulated and included an acid phosphatase, a cell wall invertase, a glutaredoxin, a Ras-like protein and a VQ motif family protein (24H; Fig. 2; Additional files 2, 3 and 4). The down-regulated genes encoded for proteins having a wide variety of functions, including a cinnamoyl-CoA reductase, an E3 ubiquitin-protein ligase, F-box family proteins, a vesicle-associated membrane protein, a histone deacetylase, and a galactosyltransferase family protein (Additional files 2, 3 and 4). The transmitting and base tissues shared only two genes, both down-regulated, which encoded for a replication protein A 32 kDa subunit and a signal recognition particle receptor alpha subunit family protein (Additional files 2, 3 and 4). No DEG were shared between the stigma and transmitting tissues (Fig. 2).

At 48H and 72H more wheat genes were up-regulated than down-regulated in the transmitting (48H - 397 up/69 down and 72H - 225 up/84 down) and base tissues (48H - 789 up/160 down and 72H - 1637 up/760 down) (Fig. 2). The number of DEG increased further at 5D and 7D in both the transmitting (5D – 3089 and 7D – 4045) and base tissues (5D – 4719 and 7D – 4786) (Fig. 2), although the ratio of up- to down-regulated genes observed at 48H and 72H was reversed at these later time points, with far more DEG being down-regulated. Although the wheat ovary becomes overwhelmed by *C. purpurea* hyphal tissue at 5D and 7D, wheat genes were detected that remained up-regulated.

Specifically, 501 and 88 DEG were up-regulated in the transmitting tissue at 5D and 7D, respectively, while 336 and 184 genes were up-regulated in the base tissue at 5D and 7D. A large percentage of these up-regulated genes belonged to functional categories related to defence and hormone pathways. At 5D 24.75% of the up-regulated genes were defence-related and 6.19% were hormone-associated in transmitting tissue, while in the base tissue 23.51% of up-regulated genes were defence-related and 4.46% were hormone-associated. At 7D 38.64% of the up-regulated genes in the transmitting tissue were defence-related and 3.41% were hormone-associated, while 40.76% were defence-related and 3.80% hormone-associated in the base tissue.

### Differential expression of hormone-associated wheat genes

**Fig. 3:**
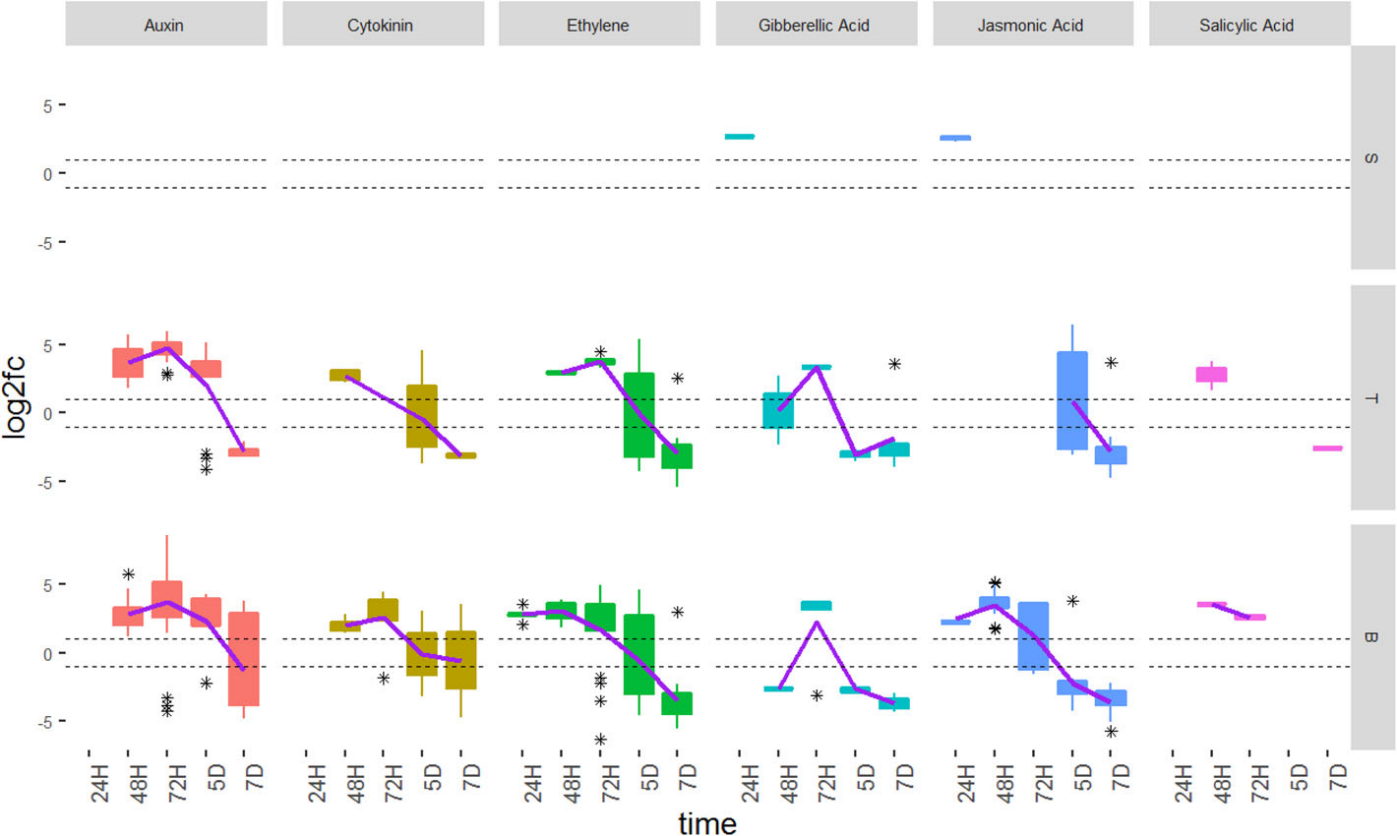
Hormone-associated differentially expressed genes (DEG) identified across time points and female floral tissues. Each box shows the number of DEG belonging to each hormone group expressed in stigma (S), transmitting (T) and base (B) tissues at 24H, 48H, 72H, 5D and 7D after inoculation with *Claviceps purpurea*, H = hours. D = days. The asterisks show the outliers beyond the upper and lower quantiles. The solid line is a regression line fitted to the data. The dotted line represents the fold change at − 1 and + 1, with genes considered not to be significantly differentially expressed if their fold change values fall between the dotted lines

**Fig. 4:**
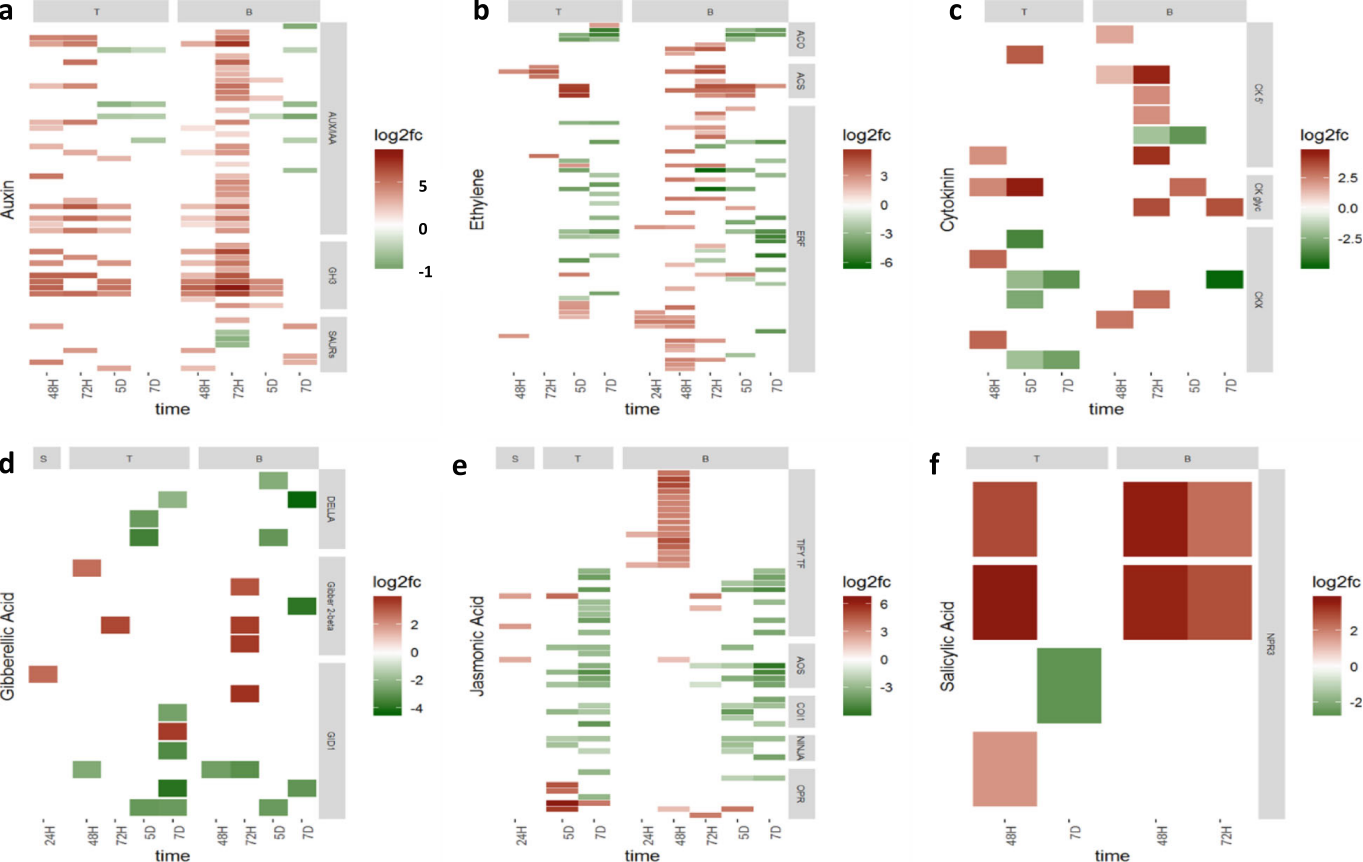
Heatmaps of hormone-associated differentially expressed genes (DEG) across time points and tissues. Each figure shows the hormone-associated genes differentially up-regulated (log2 fold change in red) or down-regulated (in green) in wheat inoculated with *Claviceps purpurea*, relative to Mock-inoculated wheat, in stigma (S), transmitting (T) and base (B) tissues of the wheat ovary, at timepoints after inoculation: 24 h (24H), 48 h (48H), 72 h (72H), 5 days (5D) and 7 days (7D). DEG are defined by functional categories. **a** Auxin-related genes ((Categories from top to bottom: Auxin/indole-3-acetic acid (AUX/IAA), Glycoside Hydrolase 3 (GH3), small Auxin-Up RNAs (SAURs)); **b** Ethylene-related genes ((Categories from top to bottom: 1-Aminocyclopropane-1-carboxylate oxidase (ACO), 1-Aminocyclopropane-1-carboxylate synthase (ACS), Ethylene responsive transcription factors (ERF)); **c** Cytokinin-related genes ((Categories from top to bottom: cytokinin riboside 5′-monophosphate phosphoribohydrolase (CK 5′), cytokinin specific glycosyltransferases (CK glyc), cytokinin oxidase/dehydrogenase (CKX)); **d** Gibberellic acid-related genes ((Categories from top to bottom: DELLA, gibberellin 2-beta-oxidase (Gibber 2-beta), GA-INSENSITIVE DWARF1 (GID1)); **e** Jasmonic acid-related genes ((Categories from top to bottom: TIFY transcription factors (TIFY TF), allene oxide synthase (AOS), coronatine-insensitive 1 (COI1), Novel INteractor of JAZ (NINJA), 12-oxophytodienoate reductase (OPR)); and **f** Salicylic acid-related genes ((Categories: NON-EXPRESSOR OF PR3 (NPR3))

Many of the wheat genes differentially transcribed in response to *C. purpurea* infection were involved in biosynthesis and signaling pathways of plant hormones, and included the ET, auxin, cytokinin, gibberellic acid (GA), salicylic acid (SA) and jasmonic acid (JA) biosynthetic and signaling pathways (Figs. 3 and 4). A list of all hormone-associated genes that were found to be differentially expressed are shown in Additional file 1 (Tables S2, S3 and S4). Hormone-associated genes were first detected in the stigma and base tissues at 24H, but not in the transmitting tissue. DEG associated with GA and JA pathways were seen in stigma tissue and JA and ET pathways in base tissue at 24H, indicating not only a very rapid induction of hormone-associated gene transcription in response to *C. purpurea* infection, but a long-distance triggering of hormone-associated gene expression in the base tissue, prior to arrival of fungal hyphae. By 48H DEG were seen in both transmitting and base tissues associated with most major groups of plant hormones. Genes were increasingly up-regulated with time, generally reaching a peak between 48H and 72H, followed by down-regulation at 5D and 7D, in transmitting and base tissues. The exception being JA-associated genes, which were not detected as differentially expressed in transmitting tissue until 5D (Figs. 3 and 4).

Within each hormone class certain genes were of particular interest. At 48H and 72H, when fungal hyphae have reached the ovule and surrounded the ovule, respectively, an up-regulation of auxin genes was observed in both the transmitting and base tissues (Fig. 4a; Additional file 5a). These genes primarily belonged to the AUX/IAA and IAA-amido synthetase (GH3) gene families, with GH3s showing particularly prolonged up-regulation in some cases, even at the 5D timepoint. AUX/IAA genes encode known transcriptional repressors of auxin response genes, while the GH3 family of genes encode auxin-conjugating enzymes that regulate the auxin pool through negative feedback. Both AUX/IAAs and GH3s are early auxin response genes, with auxin modulating their levels to re-equilibrate the system at different steady states, depending on the auxin concentration [35]. The up-regulation of these genes therefore points towards the presence of elevated levels of auxin in the floral tissues during *C. purpurea* infection, as well as a potential increase in auxin signaling. The up-regulation of the NPR3 receptor of auxin’s mutually antagonistic hormone SA was also seen in transmitting and base tissues at 48H and 72H (Fig. 4f; Additional file 5f).

Among the ET genes the two classes of genes that showed the highest up-regulation were 1-Aminocyclopropane-1-carboxylate synthase (ACS) and 1-Aminocyclopropane-1-carboxylate oxidase (ACO) (Fig. 4b; Additional file 5b). These form multi-gene families and encode ET biosynthesis enzymes, forming the final steps in the biosynthetic pathway [36]. Up-regulation was observed in both the transmitting and base tissues. ACS genes remained up-regulated at 5D, with one gene in the base tissue remaining up-regulated at the 7D. ET responsive transcription factors (ERF), which drive many of the signaling cascades in response to ET [37], were also found to be up-regulated across the transmitting and base tissues. The majority of these ERF sustained up-regulation across early time-points in the base tissue, with down-regulation occurring only at 5D and 7D. The up-regulation of genes found in both the biosynthetic and signaling pathways of ET suggest the activation of ET dependent responses during *C. purpurea* infection.

Another hormone group that responded to *C. purpurea* infection were the cytokinins (Fig. 4c; Additional file 5c). Three functional gene classes involved in cytokinin homeostasis were of interest. Firstly, cytokinin specific glycosyltransferases were observed to be up-regulated in the transmitting and base tissues throughout infection, with the up-regulation persisting even in the later time-points (5D and 7D) when the fungal hyphae have overwhelmed the ovule. These glycosyltransferases operate by deactivating cytokinin through conjugation with a sugar moiety [38]. A second gene class, also involved in cytokinin deactivation, were the cytokinin oxidase/dehydrogenase (CKX) which catalyse the irreversible degradation of cytokinins [38]. However, contrary to cytokinin glycosyltransferase, CKX were not up-regulated across all time-points, being up-regulated at 48H in the transmitting tissue, and at 48H and 72H at the base tissue. Finally, the LOG genes, encoding for cytokinin riboside 5′-monophosphate phosphoribohydrolase (CK 5′) [39], which are responsible for the single step activation of cytokinins were, in most cases, up-regulated early on during *C. purpurea* infection (48H and 72H), with the majority of up-regulated genes being detected in base tissue. The differential expression of genes involved in cytokinin homeostasis therefore suggests a significant alteration in cytokinin levels of *C. purpurea* infected female floral tissues.

Differential gene expression analyses also indicated that GA pathways were induced during infection (Fig. 4d; Additional file 5d). The gibberellin 2-beta-oxidase (GA2ox) gene was found to be up-regulated very early during infection, being found at 24H in the stigma, at 48 and 72H in the transmitting and at 72H in base tissue. GA2ox is involved in GA catabolism and inactivation of GAs and is up-regulated in response to elevated GA signaling and GA treatment [40]. The GA receptor GID1 (GA-INSENSITIVE DWARF1) gene was also up-regulated at 24H in stigma tissue, then down-regulated in transmitting and base tissues at 48H and 72H. GID1 has previously been found to be up-regulated under conditions of GA deficiency, or DELLA accumulation [41]. Taken together these findings could indicate a response by wheat to remove GA from the floral tissues.

A number of genes involved in the biosynthesis and signaling pathways of JA were also differentially expressed (Fig. 4e; Additional file 5e). With regards to the biosynthetic pathway, 12-oxophytodienoate reductase (OPR) and allene oxide synthase (AOS), which catalyses the first step in JA biosynthesis, were both found to be differentially expressed in response to *C. purpurea* infection. While OPR was up-regulated between 48H and 7D in the transmitting and base tissues, only in the case of one AOS gene was up-regulation observed at 24H in stigma and 48H in base tissue, the remaining AOS encoding genes being down-regulated. With respect to JA signaling two functional gene classes were of interest. Firstly, the F-box protein coronatine-insensitive 1 (COI1) was found to be down-regulated across the transmitting and base tissues during the last two time-points. In the presence of JA COI1 binds to jasmonate ZIM domain (JAZ) proteins leading to their ubiquitin-dependent degradation [42]. JAZ proteins repress transcription of JA-responsive genes [23], so removal of COI1 would potentially limit JAZ protein degradation and allow continued suppression of transcription of JA-responsive genes. The second signaling component affected by *C. purpurea* infection were transcription factors containing the TIFY domain. TIFY transcription factors are found in the JAZ family [43]. TIFY transcription factors were found that were up-regulated at 24H in the stigma, as well as in the base tissue. Furthermore, these transcription factors were up-regulated at 48H and 72H in base tissue. These observations suggest the possible repression of JA signaling in response to *C. purpurea* infection.

### Differential expression of defence-related wheat genes

**Fig. 5:**
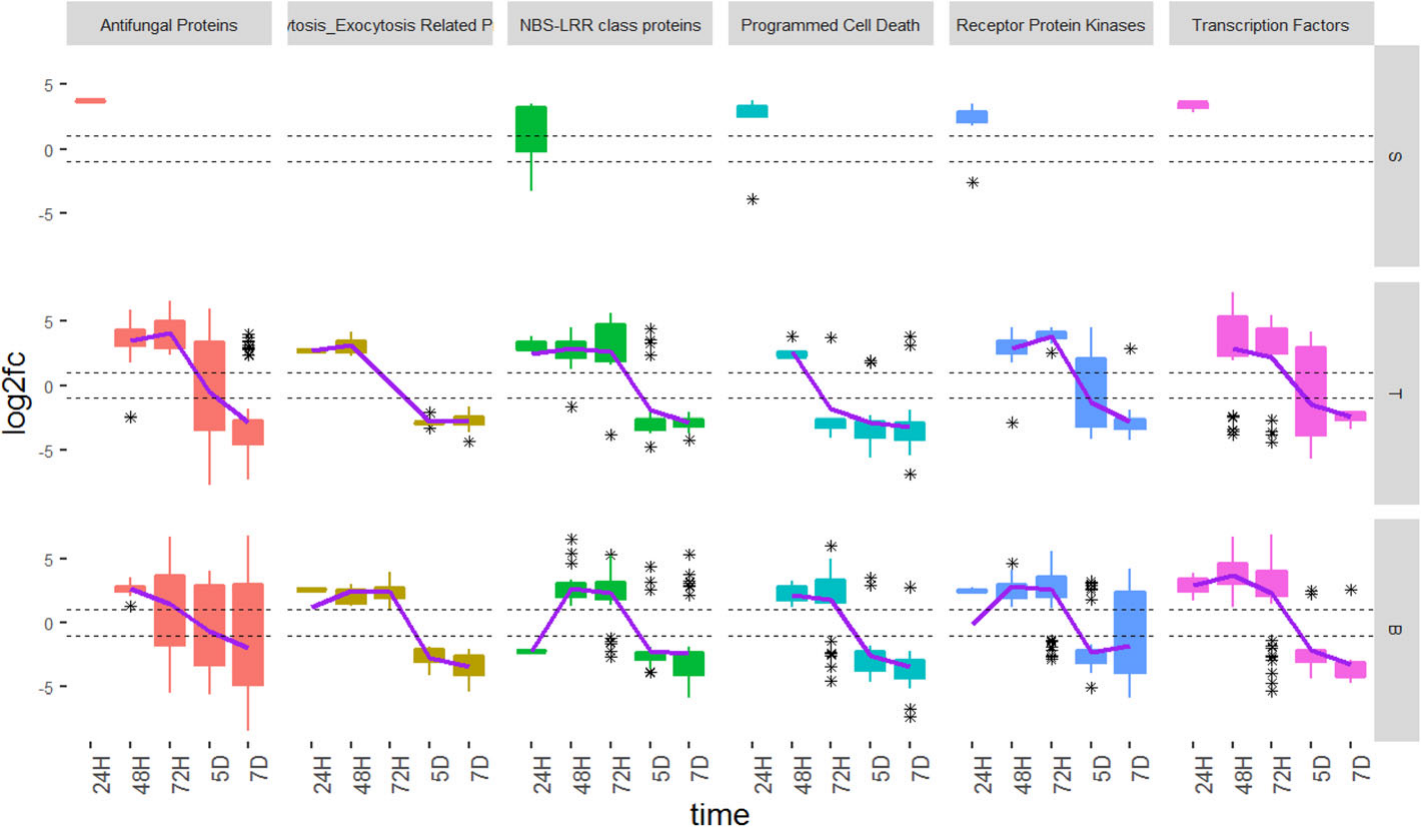
Defence-related differentially expressed genes (DEG) identified across time points and female floral tissues. Each box shows the number of DEG belonging to each defence-related functional category expressed in stigma (S), transmitting (T) and base (B) tissues at 24H, 48H, 72H, 5D and 7D after inoculation with *Claviceps purpurea*, H = hours. D = days. The asterisks show the outliers beyond the upper and lower quantiles. The solid line is a regression line fitted to the data. The dotted line represents the fold change at − 1 and + 1, with genes considered not to be significantly differentially expressed if their fold change values fall between the dotted lines

Several defence-related genes were among the wheat genes differentially expressed in response to *C. purpurea* infection. A full list can be found in Additional file 1 (Tables S5, S6 and S7). The predicted functions of these DEG were quite varied, ranging from transport and signaling, to genes involved in a wide array of metabolic reactions. Out of all the functional categories that were identified, six categories; antifungal proteins, endocytosis/exocytosis-related proteins, NBS-LRR class proteins, genes involved in programmed cell death, receptor protein kinases and transcription factors were selected as the most biologically relevant, as well as those exhibiting the most significant patterns of differential expression. Defence-related DEG were first detected at 24H and in all three ovary tissues (Fig. 5). Thus, similar to hormone-associated genes, defence-related genes were observed to be differentially expressed in base tissues prior to the colonisation of these tissues by the fungus. In general, an up-regulation of DEG in all functional categories was seen between 24H and 72H in all tissues, followed by down-regulation at 5D and 7D.

**Fig. 6:**
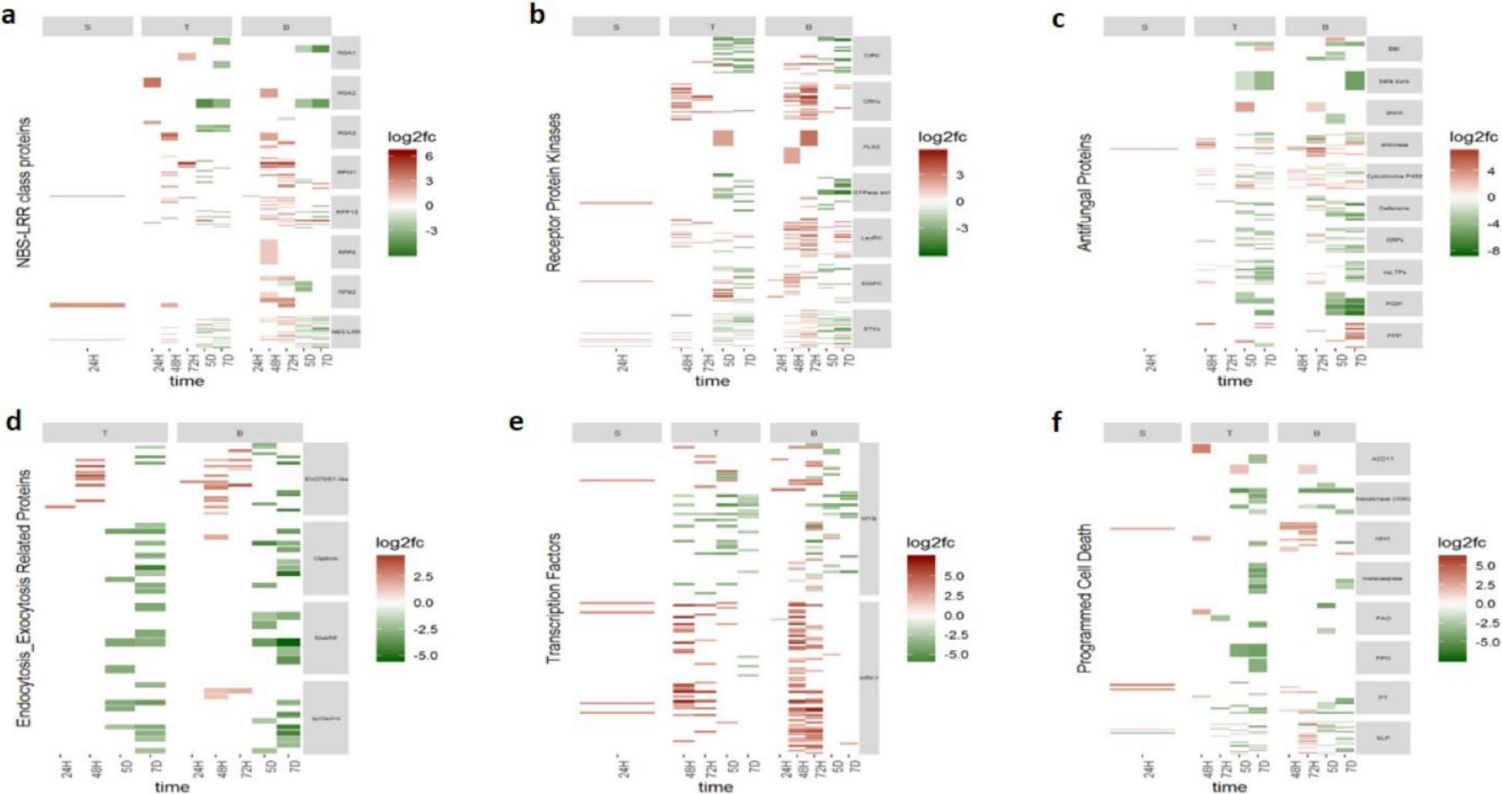
Heatmaps of defence-related differentially expressed genes (DEG) across time points and tissues. Each figure shows the defence-related genes differentially up-regulated (log2 fold change in red) or down-regulated (in green) in wheat inoculated with *Claviceps purpurea*, relative to Mock-inoculated wheat, in stigma (S), transmitting (T) and base (B) tissues of the wheat ovary, at timepoints after inoculation: 24 h (24H), 48 h (48H), 72 h (72H), 5 days (5D) and 7 days (7D). DEG are defined by functional categories. **a** NBS-LRR class proteins (functional categories from top to bottom: RGA1, RGA2, RGA3, RPM1, RPP13, RPP8, RPS2, NBS-LRR); **b** Receptor protein kinases ()functional categories from top to bottom: CBL-interacting protein kinases (CIPK), Cysteine-rich receptor-like kinases (CRKs), Flagellin-sensing 2 (FLS2), GTPase activating 1, Lectin receptor kinases (LecRK), Mitogen-activated kinase (MAPK), serine/threonine kinases (STKs)); **c** Antifungal proteins ((functional categories from top to bottom: Bowman-Birk type trypsin inhibitor (BBI), beta purothionins, chitin elicitor-binding, chitinase, Cytochrome P450, Defensins, Glycine-rich proteins (GRPs), non-specific lipid transfer proteins (nsLTPs), polygalacturonase inhibiting protein (PGIP), plant-pathogenesis proteins (PPP)); **d** Endocytosis/Exocytosis related proteins; **e** Transcription factors; and **f** Programmed cell death related genes ((functional categories from top to bottom: Accelerated Cell Death 11 (ACD11), hexokinase (HXK), Harpin induced protein (HIN1), metacaspase, polyamine oxidase (PAO), polyphenol oxidase (PPO), Potassium transporter (PT), subtilisin-like proteases (SLP))

Within the functional category NBS-LRR genes, most genes exhibited similar patterns of differential expression (Fig. 6a; Additional file 6a). Interestingly, at 24H in the base tissue NBS-LRR genes were down-regulated. These genes were then up-regulated at 48H in the transmitting and base tissues, maintaining that status until 72H, after which down-regulation was observed. However, some NBS-LRR genes exhibited LogFC values of up-regulation much higher than the rest. These genes were identified as being members of the RPM1 and RGA3 gene classes which have been found to play important roles in hypersensitive resistance [44].

Other receptor protein kinases (RPK), grouped separately from the NBS-LRR class of proteins, showed up-regulation in response to *C. purpuerea* infection (Fig. 6b; Additional file 6b). Genes resembling RPK were first up-regulated at 24H in the stigma and base tissues, being continuously up-regulated in the transmitting and base tissues until 72H. While the most abundant genes in this category were serine/threonine kinases (STK), the two classes of RPK that showed the highest levels of up-regulation were the cysteine-rich receptor-like kinases (CRK) and lectin receptor kinases (LecRK). Their up-regulation was sustained until 5D, with certain members in each class maintaining up-regulation at 7D within the base tissue. These two classes of RPK play a variety of roles in plants, including roles in down-stream signaling during pathogen recognition [45].

Genes associated with antifungal activity were induced during *C. purpurea* infection, first showing up-regulation at 24H in stigma tissue (Fig. 6c; Additional file 6c). Most genes were up-regulated in the transmitting and base tissues at the 48H and 72H timepoints. The antifungal gene classes that showed the greatest up-regulation were cytochrome P450s and chitinases. Cytochrome P450s represent one of the largest super-families of proteins in plants and are responsible for catalysing the oxygenation of many fatty acids [46]. Many of the compounds resulting from these reactions have been found to have antifungal properties [46, 47]. The chitinase encoding genes displayed the most sustained up-regulation throughout *C. purpurea* infection, across all three tissues. Chitinases are responsible for catalysis of the hydrolytic cleavage of specific bonds found in chitin, and thus play a significant role in plant defence against a range of pathogens [47].

A particularly interesting functional category of defence-related DEG were those involved in endocytosis/exocytosis processes, showing an early induction in the transmitting and base tissues (Fig. 6d; Additional file 6d). DEG in this class included SNARE proteins, syntaxins and a homologue of the exocyst complex component EXO70B1, all of which have been found to have a role in cell wall apposition formation [48]. Of these, the group that showed the highest levels of up-regulation were those of the exocyst complex component EXO70B1.

The transcription factors differentially expressed included WRKY and MYB transcription factors that were up-regulated by *C. purpurea* infection between 24H and 72H, in all three tissues (Fig. 6e; Additional file 6e). The genes identified as WRKY-type transcription factors in particular showed high levels of up-regulation. The WRKY and MYB transcription factor families have both been implicated in transcriptional reprogramming associated with plant defence responses [18].

Genes classified as involved in programmed cell death were up-regulated early, being seen in stigma tissue at 24H, peaking in transmitting tissue at 48H, and base tissue between 48H and 72H, after which these genes were down-regulated (Fig. 6f; Additional file 6f). Genes in this class included the harpin induced protein 1 (HIN1) and subtilisin-like proteases (SLP) [49, 50]. HIN1 has been found to be highly induced during proteasome-mediated programmed cell death [49], while subtilisin-like proteases have been implemented in pathogen recognition and in triggering the hypersensitive response [51].

## Discussion

Ergot has serious consequences for cereal grain quality and yield, but also directly impacts on human health due to the high levels of toxic alkaloids found in sclerotia. During the Middle Ages ergot alkaloids were responsible for the human disease known as St Anthony’s fire. While sclerotia can now be removed from contaminated grain loads by physical cleaning methods: colour sorting and gravity tables [4–6], we know very little about the interactions that occur between wheat and *C. purpurea* at a cellular and molecular genetic level. Using an RNASeq approach we report the first examination of the reprogramming of the wheat transcriptome in response to *C. purpurea* infection in defined tissues of the ovary, i.e. the stigma, transmitting and base tissues (Fig. 1).

Infection with *C. purpurea* resulted in major changes in expression of wheat genes associated with hormone metabolism and signalling, as well as a wide range of genes related to defence. There is considerable evidence which indicates the crucial role plant hormones play in the regulation of immune responses to pathogens [52]. Complex synergistic and antagonistic interactions provide the plant with the regulatory potential to activate, and fine-tune defences [52]. Our results suggest that *C. purpurea* is also able to rapidly alter hormone levels *in planta*, co-opting the host’s hormone homeostasis and/or signalling mechanisms in order to facilitate infection.

Auxin-related genes were particularly abundant among the hormone-associated genes differentially expressed in this study. Specifically, genes belonging to the AUX/IAA and IAA-amido synthetase (GH3) gene families were up-regulated during the early stages of *C. purpurea* infection. Up-regulation of these families of auxin-related genes was observed in rye ovules infected with *C. purpurea* [53]. As *C. purpurea* is able to produce and secrete significant amounts of auxin [54], it has been suggested that the pathogen co-opts its host’s auxin homeostasis in order to facilitate infection [55]. It is therefore possible that the repression of auxin signaling, through the up-regulation of AUX/IAA gene expression, and the conjugation of excessive auxin by GH3 proteins, is a direct response of the host to the elevated auxin levels produced by *C. purpurea*. Over-expression of GH3 has also been shown to result in elevated accumulation of SA [55]. While the observed up-regulation of the SA receptor NPR3, a low affinity SA receptor which requires high levels of SA to be induced [56], would support the elevation of SA within the wheat ovaries.

SA plays a crucial role in the activation of defence responses against biotrophic and hemi-biotrophic pathogens, with SA insensitive mutants showing increased susceptibility to both groups of pathogens [57]. It has also been suggested that SA acts in an opposing manner to auxin. SA can inhibit pathogen growth through the stabilisation of AUX/IAA auxin repressors, achieved by limiting the auxin receptors required for their degradation [58]. Indeed, our data show the down-regulation of an auxin binding protein (probably an auxin receptor) within the transmitting and base tissues, which coincides with the up-regulation of the AUX/IAA genes.

The ET and JA biosynthetic genes, *ACS* and *ACO*, and *OPR* and *AOS*, respectively, were up-regulated in transmitting and base ovary tissues upon infection by *C. purpurea*, while the JA signaling gene *COI1* was down-regulated. Infection of wheat ears with *F. graminearum*, the causal agent of FHB, also resulted in up-regulation of the JA biosynthetic genes AOS and OPR in the FHB resistant variety Wangshuibai, while the JA signaling gene *COI1* was down-regulated in the susceptible wheat upon infection with *F. graminearum* [59]. Similar patterns in the expression of ET genes, namely the up-regulation of the ET biosynthetic genes ACS and ACO were also observed by [59, 60]. Up-regulation of ACS and ACO genes was observed in rice (*Oryza sativa*), accompanied by the enhanced emission of ET, in response to infection with the hemi-biotroph fungus *M. grisea* [61]*.* ET responsive transcription factors (ERFs) were also up-regulated during the early stages of infection. ERFs play a significant role in the regulation of defence, and changes in their expression have been shown to lead to changes in resistance to different types of fungi [62]. For instance, in *Arabidopsis*, while the constitutive expression of ERF1 enhances tolerance to *Botrytis cinereal* infection [63], the over-expression of ERF4 leads to an increased susceptibility to *F. oxysporum* [62].

Our data showed that the induction of ET biosynthesis genes *ACS* and *ACO* coincided with the induction of two genes involved in JA biosynthesis. Studies have suggested that ET signaling operates in a synergistic way with JA signaling to activate defence reactions, and in particular defence reactions against necrotrophic pathogens [64]. It has also long been considered that JA/ET signaling pathways act in a mutually antagonistic way to SA, however, other studies have shown that ET and JA can also function in a mutually synergistic manner, depending on the nature of the pathogen [65].

Cytokinins were also implicated in *C. purpurea* infection of wheat, with the up-regulation of CKX and cytokinin glycosyltransferase in transmitting and base tissues. These two cytokinin inducible genes are both involved in cytokinin homeostasis, and function by degrading and conjugating cytokinin [57]. The cytokinin glycosyltransferase deactivates cytokinin through conjugation with a sugar moiety, while CKX catalyzes the irreversible degradation of cytokinins in a single enzymatic step [66]. *C. purpurea* is able to secrete large amounts of cytokinins *in planta,* in order to facilitate infection [67], and *M. oryzae*, the rice blast pathogen also secretes cytokinins, being required for full pathogenicity [68]. The up-regulation of these cytokinin degrading wheat genes maybe therefore be in response to elevated levels of *C. purpurea* cytokinins, and a defence response of the host.

The early induction of the GA receptor GID1 in wheat stigma tissue, as well as the subsequent up-regulation of key GA catabolic enzymes, such as GA2ox, in transmitting and base tissues, suggests that GA accumulates in response to *C. purpurea* infection. The accumulation of GA likely leads to the degradation of the negative regulators of GA signaling, the DELLA proteins. This observation is in accordance with a study in which the *Arabidopsis* loss of function quadruple-*della* mutant was resistant to the biotrophic pathogens *Pst*DC3000 and *Hyaloperonospora arabidopsidis* [22]. Furthermore, a recent study identified a partial resistance to *C. purpurea* associated with the DELLA mutant, semi-dwarfing alleles, *Rht-1Bb* and *Rht-1Db* [69].

The complexity of plant immunity was further evident from the variety of genes with known roles in plant defence that were differentially expressed in response to *C. purpurea* infection. All categories of defence genes, except endocytosis/exocytosis-related genes, were up-regulated in stigma tissue at 24H. Many RPK and NBS-LRR class proteins, which are known to be involved in PAMP and effector recognition, were up-regulated early in *C. purpurea* infection, even though this wheat-*C. purpurea* interaction represented a susceptible interaction, where *C. purpurea* was able to complete its infection life cycle.

Many NBS-LRR proteins detect effector molecules produced by the pathogen, either directly, by binding with the effector protein, or indirectly through the modifications these effectors have on host target proteins [70]. The indirect mechanisms tend to operate by the NBS-LRR proteins binding to key host targets of the pathogen, and trigger defence when those targets are altered in response to infection. The up-regulation of these NBS-LRR proteins at 24H in the transmitting and base tissues, before the arrival of fungal hyphae in these tissues, suggests that these genes are induced in response to a pathogen, or plant-derived, mobile signal. The up-regulation of a wide variety of NBS-LRR proteins early during *C. purpurea* infection could indicate an attempt by the host plant to increase its recognition capacity of *C. purpurea* effectors. This would then lead to activation of specific defence reactions, such as cell wall modification, secondary metabolite production, and even programmed cell death, in order to counteract pathogen attack. Homologues of known NBS-LRR resistance (R-) genes were identified, including *RGA2* and *RGA3*, which are required for resistance to leaf rust (*Puccinia triticina*) in tetraploid and hexaploid wheat [71]. Homologues of the R-genes *RPM1* and *RPS2* have both been found to be significantly induced in response to the biotrophic fungus *Exobasidium vexans* that causes blister blight in tea [72].

In addition to the specific NBS-LRR class of RPK proteins, other RPK, namely serine/threonine kinases (STK) and cysteine-rich receptor-like protein kinases (CRK), were found to be strongly induced throughout *C. purpurea* infection. Contrary to the NBS-LRR proteins these RPKs exhibited up-regulation that was sustained at the later time-points of *C. purpurea* infection. STK are membrane proteins that form a first line of defence, recognising PAMP, which can then lead to the activation of MAPK signaling cascades and ultimately other defence-related genes [45]. CRK are a sub-member of receptor-like kinases and many genes belonging to this family of proteins have been found to be induced by a variety of pathogens. One such CRK was found to be induced in barley in response to the biotrophic fungus *Blumeria graminis f. sp. hordei*, which causes barley powdery mildew [73]. Taken together these results would suggest that wheat recognizes *C. purpurea* through the activation of multiple receptor proteins, which then trigger an array of defence responses, even in this wheat-*C. purpurea* compatible interaction.

A common, early response upon pathogen infection is cell wall modification. Cell wall defensive appositions called papillae are formed beneath the attempted pathogen penetration sites of many biotrophic and hemi-biotrophic pathogens [48]. In barley, this process has been shown to be facilitated through the action of genes such as SNARE proteins, syntaxins and the exocyst complex component EXO70B. Genetic screening of mutants which allowed increased penetration by *B. graminis* identified the crucial role of syntaxins and SNARE proteins in cell wall modification in response to attempted fungal penetration [74]. Homologues of these genes were up-regulated in wheat during the early stages of *C. purpurea* infection, although to the best of our knowledge, papillae have not been observed in cereal- *C. purpurea* interactions.

The observed induction of WRKY and MYB transcription factors during the early stages of *C. purpurea* infection further points towards the reprogramming of the wheat transcriptome. WRKY transcription factors participate in regulating defence gene expression at various levels, activating the production of antimicrobial compounds and triggering cell death, while MYB transcription factors have also been found to be involved in the induction of the hypersensitive cell death response [75, 76]. The effects of these transcription factors were evident within our dataset from the induction of genes with antifungal action, roles in cell wall modification and programmed cell death, and the generation of secondary metabolites. The observed early induction of antifungal compounds, such as chitinases and defensins, known inhibiters of fungal growth [77, 78], has also been observed during infection of rye by *C. purpurea* [53].

## Conclusions

Ergot is a serious disease of many commercial cereal crops. Contaminating sclerotia result in seed lots being down-graded for human consumption or even discarded due to the highly toxic ergot alkaloids that accumulate in sclerotia [4, 7]. The expansion of hybrid wheat and barley markets is seeing a resurgence of ergot in these open-flowering production systems [10], while recent evidence suggests that ergot alkaloids can contaminate healthy seed in situ, as they develop above and below flowers infected by *C. purpurea* [9]. Therefore, it is essential that we obtain a better understanding of the *C. purpurea* infection process in important cereal crops such as hexaploid wheat. Our data suggests that upon infection by *C. purpurea* the wheat plant activates several defence mechanisms at early stages of infection. In addition to well characterised defence-related genes, wheat genes involved in hormone homeostasis and signaling pathways were induced. These hormone-associated genes may be up-regulated as part of a defence response on the part of the host, but equally could be induced by *C. purpurea* to create an environment suitable for *C. purpurea* colonisation and reproduction, and to disrupt the host’s defence responses. The evidence for a long-distance mobile signal triggering differential gene expression at the base of the ovary, long before the arrival of the pathogen, could equally be derived from *C. purpurea*, sent to prepare the basal tissue for arrival of fungal hyphae, or be plant-derived and sent from the infected stigma to trigger a systemic defence reactions.

## Methods

### Plant material, *Claviceps purpurea* inoculations and sampling

A cytoplasmic male sterile hexaploid wheat line developed at NIAB by Steve Bentley (personal communication) was used in all *C. purpurea* inoculations. Plants were grown in the glasshouse at an18^o^C/16 h day and 15 °C/8 h night cycle, supplemented when needed by artificial light at 240 μmol m^− 2^ s^− 1^. The middle florets of each ear, of the first tiller, were inoculated with a single *C. purpurea* isolate when the stigma became receptive (i.e. fluffy in appearance), as described in [69]. *C. purpurea* inoculations were carried out using a 2 ml syringe and fine needle, delivering the conidia suspension between the lemma and palea of each floret. Twelve florets were inoculated on each ear. Each inoculated ear represented a single replicate, with five replicates being collected for each time point and tissue sampled. Mock-inoculated florets were injected with ultra-pure water. *C. purpurea (Cp)*- and Mock-inoculated samples were taken at 10 min, 1, 5, 24, 48 and 72 h (H), and 5 and 7 days (D) after inoculation for both microscopy and RNASeq analyses (Table 2).

The *C. purpurea* UK isolate 04–97.1 [79] were used in all inoculations. Isolate 04–97.1 was recovered from long-term glycerol stocks kept at -80 °C by inoculation onto the male sterile line two weeks prior to conidia being required. Fresh conidia, in the form of honeydew, were collected and diluted in ultra-pure water to a concentration of 1 × 10^− 6^ spores ml^1^. These conidia were used to inoculate plants over a 3-day period, being kept at 4 °C.

Additional fungal samples were collected including replicates of conidia from honeydew, and mycelia of *C. purpurea.* Conidia from a single inoculated ear was collected 10–12 days after inoculation and was resuspended in 1 ml distilled water. Spores were centrifuged at 6000 rpm and then resuspended in 50 ml RNA*later* (supplied by Thermofisher scientific) Mycelial samples had been grown for 24 h in liquid Mantle media at 20 °C before collection by centrifugation and resuspension in 50 ml RNA*later* and stored at -80 °C. RNA was extracted for RNASeq analyses from both *C. purpurea* mycelia and conidia.

### Preparation of floral tissues for microscopy and RNA extraction

Whole ovaries were removed from each inoculated floret and sectioned using a double edge razor that had been wiped with RNAseZap (supplied by Thermofisher scientific). A longitudinal section was made along the dorsal groove of each ovary allowing for easy identification of the stigma, transmitting and base tissues (Fig. 1). Half of the ovary was placed into formaldehyde for fixing and subsequent epifluorescent and confocal microscopy. The other half was placed into 30 μl of RNA*later* and left for 24 h to allow full penetration of the liquid.

### Microscopy procedures

Ovary halves reserved for microscopy were stained with a solution of 0.05% aniline blue in potassium phosphate buffer, pH 9.0. Ovaries were examined using epifluorescence microscopy and scored for the presence of stained hyphae in stigma, transmitting and base tissues, at each of the time points. For high resolution confocal microscopy ovary halves were fixed in 1 M KOH for 24 h, rinsed in water, and then treated with 0.3 mg/ml amylase for 36–48 h at 37 °C. Ovaries were stained using the mPS-PI technique [80]. Ovaries were treated with Schiff reagent (100 mM sodium metabisulphite and 0.15 M HCL) and 100 μg/ml propidium iodide for 1–2 h at room temperature, rinsed in water, and then stained and cleared in a modified SCALE solution with aniline blue [81]; 50 mM K_2_HPO_4_, 4 M Urea, 10% glycerol, 0.1% Triton X-100 and 0.05% aniline blue; pH 9.0). Ovaries were mounted in staining solution and imaged with a Leica SP5 confocal microscope (Leica Microsystems UK Ltd). Aniline blue-stained tissues were visualised using an excitation of 405 nm and detected at 415–490 nm and propidium iodide was visualised using an excitation of 561 nm and detected at 575–720 nm.

### RNA extraction, library construction and RNAseq

The individual ovary halves (up to 12 ovaries halves per ear) collected from each *Cp*-inoculated ear were pooled if the corresponding ovary half was shown by microscopy to be infected with *C. purpurea* infection. The half ovaries from one ear formed an RNA replicate. Each ovary half was sectioned into stigma, transmitting and base tissue. Tissue disruption of plant and fungal tissues was carried out using 2 mm RNase-free steel balls (Spheric Transfer). RNA was prepared using the Trizol (Invitrogen) method. RNA was DNase treated (Qiagen) and then cleaned using RNeasy 96-well columns, before quantification using nanodrop. RNA integrity was assessed using a Shimadzu MultiNA in order to select 3 of the 5 replicates RNA samples for Illumina TruSeq library preparation.

Three replicate RNAseq libraries were made of the Mock- and *Cp*-inoculated wheat ovaries for each of the three tissues - stigma, transmitting and base tissues, at each time point. For stigma viable tissue was not available beyond 24H. RNAseq libraries were also made from ungerminated *C. purpurea* conidia (two replicates) and *C. purpurea* mycelium grown in vitro (three replicates). RNAseq libraries were prepared and sequenced by Source Bioscience (www.sourcebioscience.com): mRNA was isolated using Illumina poly-T oligo-attached magnetic beads, undergoing two rounds of purification. The mRNAs were fragmented and primed with random hexamers for cDNA synthesis. Libraries were prepared in accordance with the Illumina TruSeq RNA sample preparation guide (November 2010, rev. A) for Illumina Single-End Multiplexed Sequencing. Libraries were pooled and run on two flow cells.

### Bioinformatics pipeline: pre-processing

Quality checking of fastq files was performed using FastQC [82]. Adapter sequences were removed using FASTX clipper [83] (parameters: –M 15 –l 20 –a < adapter sequence>). Sequence ends with quality scores of less than 20 were trimmed and sequences shorter than 35 were removed (parameters: –t 20 –l 35) using FASTQ Quality Trimmer [83].

### Genome-guided assembly

The bread wheat variety Chinese Spring (IWGSC RefSeq v1; URGI INRA) cDNA version 1 and the *C. purpurea* cDNA (Ensembl release 35) were merged to form a transcriptome fasta reference sequence. Both Mock- and *Cp*-inoculated reads were aligned against this indexed reference sequence using bowtie2 with the default parameters [84]. Using SAMtools alignment files were converted in binary format (command: view –b) [85] and reads with low mapping quality (option: view –b –q 5) and PCR duplicates (option: rmdup) were removed [53] Percentage alignment results are provided in Additional file 1 (Table S1). The average proportion of reads removed across all libraries was 0.0093%.

### Cross-mapping check

As the pipeline involved the merging of the wheat IWGSC RefSeq v1 and *C. purpurea* cDNA reference (Ensembl release 35) sequences we checked whether there was reciprocal mapping of reads between the wheat and *C. purpurea* transcriptomes. We mapped all Mock-inoculated wheat sample reads to the *C. purpurea* reference sequence. Likewise, we mapped *C. purpurea* reads (two reps of conidia-only and two reps of media-grown *C. purpurea* mycelium) to the wheat transcriptome reference sequence. Removal of low-quality reads and mapping were performed as described above. After removal of low-quality reads and PCR duplicates, we calculated the percentage alignment of wheat reads mapping to the *C. purpurea* transcriptome and the *C. purpurea* reads mapping to the wheat transcriptome reference sequences.

### De novo assembly of unmapped *Claviceps purpurea* reads

The percentage alignment of reads mapping to the combined wheat-*C. purpurea* reference transcriptome dropped at the 5D and 7D timepoints, which we speculated was due to a lack of coverage within the *C. purpurea* cDNA (Ensembl release 35) reference transcriptome. To generate a *C. purpurea* reference transcriptome more suited to the isolate used in this study we performed de novo assembly using Trinity. Reads from the ungerminated *C. purpurea* conidia (2 reps) and *C. purpurea* grown on artificial media (3 reps) libraries were mapped to the *C. purpurea* cDNA (Ensembl release 35) transcriptome references. Unmapped reads were extracted using SAMtools (command: view -b -f 4). Read duplicates were tagged and removed using GATK (option: MarkDuplicates [86]; and PRINSEQ (option: derep) [87] respectively. This aimed to reduce memory space and increase calculation speed. This resulted in 1.33 M reads in fastq format. Trinity was used to perform de novo assembly using the default kmer length equivalent to 25 (options: --bflyHeapSpaceMax –bflyHeapSpaceInit –bflyCalculateCPU). After assembly, contigs with no predicted open-reading frame (ORF) were dropped using a web-based ORF predictor [88]. *C. purpurea* cDNA (Ensembl release 35) and the de novo assembled references were merged to form a new *C. purpurea* reference transcriptome. Reads from all the wheat-*C. purpurea* libraries were remapped to the combined wheat IWGSC RefSeq v1 and new *C. purpurea* reference transcriptome.

### Read count quantification and differential gene expression analysis

Quantification of read counts contained within the alignment bam files was performed using Salmon’s alignment-based mode (parameters: --biasCorrect --useErrorModel) [89]. The annotation name and the number of reads columns generated by Salmon were extracted and a count data matrix created using R (in Linux). Rows with low read counts (R command: rowSums (CD@data) > ncol (CD)) were removed to reduce object size and increase calculation speed. Histograms were created before and after the removal of near-zero read counts or low expressed isoforms to assess the distribution curve of the datasets.

To normalize datasets with respect to library size, library scaling factors were calculated using baySeq trimmed mean of M-values (TMM) [90]. MA plots (where M is the difference in log expression values and A is the average [91]; were created and used to determine if the normalization procedure was adequate with respect to library size. Loess regression curves [92] were plotted to determine whether the normalization step had “centered” the MA plots.

Pairwise, cross-conditional differential gene expression analysis between *Cp-* and Mock-inoculated samples was performed using baySeq [93–95]. The average normalized read counts of all replicates of each tissue by time point sample were calculated, incremented by 1 to avoid 0 denominators in subsequent analyses. Expression ratios were obtained by dividing the average normalized counts of the *Cp*- over the Mock-inoculated samples (Treatment/Control or T/C), generating log (base 2) ratio or fold changes (FC). Genes are considered to be statistically differentially expressed between *Cp-* and Mock-inoculated treatments when they exhibit a FC ≥ 2 (or |log_2_FC| ≥1) at a false discovery rate (FDR) *p*-value correction < 0.05, and showed an absolute difference > 10 [96].

Customised heatmaps and boxplots were produced using R to visualise gene expression across tissues and time points. Fitted regression lines were superimposed onto the boxplots to facilitate interpretation of gene expression patterns across time.

### Annotation of differentially expressed genes

The genes that were found to be differentially expressed in the stigma, transmitting or base tissues, at one or more time points, were annotated using Blast2go (http://www.blast2go.com/b2ghome). For functional annotation the genes were aligned against the National Centre of Biotechnology Information (NCBI) nr protein database. The blastx function was used to search gene sequences against the Swiss-Prot protein database, with the e-value cut-off set at 1e^− 5^. Gene names were assigned based on the top Blastx hit, having the highest similarity score. Genes related to hormone pathways, defence and photosynthesis were further, manually explored based on the names that had been assigned to them during annotation. The functions of these potential genes of interest were investigated through manual searches of scientific literature databases.

## Supplementary Information

**Additional file 1: Fig. S1**: MA plots for wheat transcripts at 10 mins, 1 h and 24 h. Loess curves (red/blue) were drawn along with the line of symmetry at M = 0 (yellow). The blue Loess curve are smoothened curves set at family = “symmetric”. The red is a regular Loess curve (M ~ A). In some figures, only one line is visible since two/all curves may overlap. **Fig. S2**: MA plots for wheat transcripts at 48 h, 72 h, 5 days, and 7 days. Loess curves (red/blue) were drawn along with the line of symmetry at M = 0 (yellow). The blue Loess curve are smoothened curves set at family = “symmetric”. The red is a regular Loess curve (M ~ A). In some figures, only one line is visible since two/all curves may overlap. To demonstrate the asymmetric distribution of points, MA plots were generated using both wheat (blue arrow) and *C. purpurea* (red) transcripts. **Table S1**: Percentage alignment rates of pair-end reads from 114 Mock and Cp-inoculated libraries against the International Wheat Genome Sequencing Consortium (IWGSC) wheat genomic reference from wheat variety Chinese Spring [30]. **Table S2**: Table of all hormone-associated genes differentially expressed in base tissue. Red represents up-regulated genes and green down-regulated genes. A schematic representation of the stages of *Claviceps purpurea* development in the wheat ovary at each time point is shown at the top of the table. **Table S3**: Table of all hormone-associated genes differentially expressed in transmitting tissue. Red represents up-regulated genes and green down-regulated genes. A schematic representation of the stages of *Claviceps purpurea* development in the wheat ovary at each time point is shown at the top of the table. **Table S4**: Table of all hormone-associated genes differentially expressed in stigma tissue. Red represents up-regulated genes and green down-regulated genes. A schematic representation of the stages of *Claviceps purpurea* development in the wheat ovary at each time point is shown at the top of the table. **Table S5**: Table of all defence-related genes differentially expressed in stigma tissue. Red represents up-regulated genes and green down-regulated genes. A schematic representation of the stages of *Claviceps purpurea* development in the wheat ovary at each time point is shown at the top of the table. **Table S6**: Table of all defence-related genes differentially expressed in transmitting tissue. Red represents up-regulated genes and green down-regulated genes. A schematic representation of the stages of *Claviceps purpurea* development in the wheat ovary at each time point is shown at the top of the table. **Table S7**: Table of all defence-related genes differentially expressed in base tissue. Red represents up-regulated genes and green down-regulated genes. A schematic representation of the stages of *Claviceps purpurea* development in the wheat ovary at each time point is shown at the top of the table.

**Additional file 2 **Wheat genes differentially expressed in stigma tissue at time points 1 and 24 h after inoculation with *Claviceps purpurea*.

**Additional file 3 **Wheat genes differentially expressed in transmitting tissue at time points 24, 48 and 72 h, and 5 and 7 days after inoculation with *Claviceps purpurea*.

**Additional file 4 **Wheat genes differentially expressed in base tissue at time points 24, 48 and 72 h, and 5 and 7 days after inoculation with *Claviceps purpurea*.

**Additional file 5 **Heatmaps of hormone-associated differentially expressed genes (DEG) across time points and tissues. DEG are defined by functional categories: Each figure shows the hormone-associated genes differentially up-regulated (log2 fold change in red) or down-regulated (in green) in wheat inoculated with *Claviceps purpurea*, relative to Mock-inoculated wheat, in stigma (S), transmitting (T) and base (B) tissues of the wheat ovary, at timepoints after inoculation: 24 h (24H), 48 h (48H), 72 h (72H), 5 days (5D) and 7 days (7D). Figure (a) Auxin-related genes ((Categories from top to bottom: Auxin/indole-3-acetic acid (AUX/IAA), Glycoside Hydrolase 3 (GH3), small Auxin-Up RNAs (SAURs)). Figure (b) Ethylene-related genes ((Categories from top to bottom: 1-Aminocyclopropane-1-carboxylate oxidase (ACO), 1-Aminocyclopropane-1-carboxylate synthase (ACS), Ethylene responsive transcription factors (ERF)). Figure (c) Cytokinin-related genes ((Categories from top to bottom: cytokinin riboside 5′-monophosphate phosphoribohydrolase (CK 5′), cytokinin specific glycosyltransferases (CK glyc), cytokinin oxidase/dehydrogenase (CKX)). Figure (d) Gibberellic acid-related genes ((Categories from top to bottom: DELLA, gibberellin 2-beta-oxidase (Gibber 2-beta), GA-INSENSITIVE DWARF1 (GID1)). Figure (e) Jasmonic acid-related genes ((Categories from top to bottom: TIFY transcription factors (TIFY TF), allene oxide synthase (AOS), coronatine-insensitive 1 (COI1), Novel INteractor of JAZ (NINJA), 12-oxophytodienoate reductase (OPR)). Figure (f) Salicylic acid-related genes ((Categories: NON-EXPRESSOR OF PR3 (NPR3)). Traes number refers to the gene annotation provided by the International Wheat Genome Sequencing Consortium (IWGSC) wheat genomic reference from wheat variety Chinese Spring [30].(PPTX 7966 kb)

**Additional file 6 **Heatmaps of defence-related differentially expressed genes (DEG) across time points and tissues. DEG are defined by functional categories: Each figure shows the defence-related genes differentially up-regulated (log2 fold change in red) or down-regulated (in green) in wheat inoculated with *Claviceps purpurea*, relative to Mock-inoculated wheat, in stigma (S), transmitting (T) and base (B) tissues of the wheat ovary, at timepoints after inoculation: 24 h (24H), 48 h (48H), 72 h (72H), 5 days (5D) and 7 days (7D). Figure (a) NBS-LRR class proteins (functional categories from top to bottom: RGA1, RGA2, RGA3, RPM1, RPP13, RPP8, RPS2, NBS-LRR). Figure (b) Receptor protein kinases ()functional categories from top to bottom: CBL-interacting protein kinases (CIPK), Cysteine-rich receptor-like kinases (CRKs), Flagellin-sensing 2 (FLS2), GTPase activating 1, Lectin receptor kinases (LecRK), Mitogen-activated kinase (MAPK), serine/threonine kinases (STKs)). Figure (c) Antifungal proteins ((functional categories from top to bottom: Bowman-Birk type trypsin inhibitor (BBI), beta purothionins, chitin elicitor-binding, chitinase, Cytochrome P450, Defensins, Glycine-rich proteins (GRPs), non-specific lipid transfer proteins (nsLTPs), polygalacturonase inhibiting protein (PGIP), plant-pathogenesis proteins (PPP)). Figure (d) Endocytosis/Exocytosis related proteins. Figure (e) Transcription factors. Figure (f) Programmed cell death related genes ((functional categories from top to bottom: Accelerated Cell Death 11 (ACD11), hexokinase (HXK), Harpin induced protein (HIN1), metacaspase, polyamine oxidase (PAO), polyphenol oxidase (PPO), Potassium transporter (PT), subtilisin-like proteases (SLP)). Traes number refers to the gene annotation provided by the International Wheat Genome Sequencing Consortium (IWGSC) wheat genomic reference from wheat variety Chinese Spring [30].(PPTX 9371 kb)

## Data Availability

The datasets generated and analysed during the current study are available at ArrayExpress, Accession Number E-MTAB-9799. http://www.ebi.ac.uk/arrayexpress/help/FAQ.html#cite).

## Abbreviations

ACO: 1-Aminocyclopropane-1-carboxylate oxidase
ACS: 1-Aminocyclopropane-1-carboxylate synthase
AOS: Allene Oxide Synthase
AUX/IAA: Auxin/indole-3-acetic acid
CKs: Cytokinins
CKX: cytokinin oxidase/dehydrogenase
COI1: Coronatine-Insensitive 1
CRK: Cysteine-rich Receptor-like Kinases
DEG: Differentially Expressed Genes
ERF: Ethylene responsive transcription factors
ET: Ethylene
ETS: Effector Triggered Suppression
FHB: Fusarium Head Blight
GA: Gibberellic Acid
GA2ox: Gibberellin 2-beta-oxidase
GID1: GIBBERELLIN-INSENSITIVE DWARF1
GH3: Glycoside Hydrolase 3
HIN1: Harpin Induced Protein 1
HSP40s: Heat-Shock Protein 40
IAA: Indole-3-acetic acid
JA: Jasmonic Acid
JAZ1: Jasmonate-zim-domain protein 1
LecRK: Lectin Receptor Kinases
MAPK: Mitogen-Activated Protein Kinase
NBS-LRR: Nucleotide-Binding Site Leucine-Rich Repeat
NPR3: NON-EXPRESSOR OF PR3
OPR: 12-Oxophytodienoate reductase
PAMPs: Pathogen-associated Molecular Patterns
PTI: PAMP-Triggered Immunity
QTL: Quantitative Trait Loci
ROS: Reactive Oxygen Species
RPK: Receptor Protein Kinases
SA: Salicylic Acid
SAR: Systemic Acquired Resistance
SAURs: small Auxin-Up RNAs
SLP: Subtilisin-like Proteases
STK: Serine/Threonine Kinases
