# Supplementary material for: Reprogramming of the wheat transcriptome in response to infection with Claviceps purpurea, the causal agent of ergot
Source: BMC Plant Biol. 2021 Jul 2;21:316. doi: 10.1186/s12870-021-03086-3 (PMC8252325; doi:10.1186/s12870-021-03086-3)

**Additional file 1**

**Table S1: Percentage alignment rates of pair-end reads from 114 Mock and Cp-inoculated libraries against the International Wheat Genome Sequencing Consortium (IWGSC) wheat genomic reference from wheat variety Chinese Spring [30].**

| Library | No. of reads | Percentage alignments against RefSeq | **Average coverage** |
| --- | --- | --- | --- |
| **10 min** |  |  |  |
| MockStigmaLib1 | 8338638 | 78.12 | 9.265153333 |
| MockStigmaLib4 | 8140388 | 80.08 | 9.044875556 |
| MockTTLib1 | 5342892 | 78.82 | 5.936546667 |
| MockTTLib4 | 10329694 | 83.5 | 11.47743778 |
| MockTTLib5 | 8266242 | 83.73 | 9.184713333 |
| MockBaseLib1 | 8646439 | 83.08 | 9.607154444 |
| MockBaseLib4 | 4110531 | 68.87 | 4.567256667 |
| MockBaseLib5 | 26563767 | 82.14 | 29.51529667 |
| CpStigmaLib1 | 3719359 | 78.47 | 4.132621111 |
| CpStigmaLib5 | 2565118 | 80.02 | 2.850131111 |
| CpStigmaLib6 | 5282310 | 78.49 | 5.869233333 |
| CpTTLib2 | 7106179 | 84.62 | 7.895754444 |
| CpTTLib7 | 7402362 | 82.87 | 8.224846667 |
| CpBaseLib1 | 9518508 | 84.08 | 10.57612 |
| CpBaseLib5 | 5570863 | 83.86 | 6.189847778 |
| CpBaseLib6 | 10349223 | 82.69 | 11.49913667 |
| **1 hour** |  |  |  |
| MockStigmaLib1 | 3005658 | 77.75 | 3.33962 |
| MockStigmaLib4 | 6943134 | 78.11 | 7.714593333 |
| MockStigmaLib7 | 958381 | 77.46 | 1.064867778 |
| MockTTLib1 | 3392286 | 83.05 | 3.769206667 |
| MockTTLib5 | 15227496 | 84.2 | 16.91944 |
| MockTTLib7 | 3991733 | 80.92 | 4.435258889 |
| MockBaseLib1 | 4039352 | 81.54 | 4.488168889 |
| MockBaseLib5 | 10455117 | 84.6 | 11.61679667 |
| MockBaseLib7 | 9038734 | 82 | 10.04303778 |
| CpStigmaLib2 | 1471138 | 78.01 | 1.634597778 |
| CpStigmaLib3 | 13566658 | 81.34 | 15.07406444 |
| CpTTLib2 | 10616236 | 67.8 | 11.79581778 |
| CpTTLib3 | 14022049 | 84.34 | 15.58005444 |
| CpTTLib5 | 2801902 | 81.08 | 3.113224444 |
| CpBaseLib2 | 14530662 | 81.46 | 16.14518 |
| CpBaseLib3 | 1698614 | 83.85 | 1.887348889 |
| CpBaseLib5 | 5722919 | 82.14 | 6.358798889 |
| **5 hours** |  |  |  |
| MockStigmaLib4 | 728409 | 74.87 | 0.809343333 |
| MockStigmaLib5 | 751563 | 75.17 | 0.83507 |
| MockTTLib4 | 1068529 | 80.87 | 1.187254444 |
| MockTTLib5 | 1274409 | 81.71 | 1.41601 |
| MockBaseLib4 | 4144793 | 80.54 | 4.605325556 |
| MockBaseLib5 | 1181001 | 78.29 | 1.312223333 |
| CpStigmaLib1 | 581836 | 77.63 | 0.646484444 |
| CpStigmaLib4 | 148791 | 71.69 | 0.165323333 |
| CpStigmaLib5 | 526205 | 79.66 | 0.584672222 |
| CpTTLib1 | 5894932 | 82.41 | 6.549924444 |
| CpTTLib3 | 6345375 | 82.29 | 7.050416667 |
| CpTTLib4 | 1808326 | 80.71 | 2.009251111 |
| CpBaseLib1 | 11222147 | 83.59 | 12.46905222 |
| CpBaseLib3 | 19834227 | 83.62 | 22.03803 |
| CpBaseLib4 | 2644868 | 81.83 | 2.938742222 |
| **24 hours** |  |  |  |
| MockStigmaLib1 | 12260120 | 79.1 | 13.62235556 |
| MockStigmaLib3 | 11062304 | 30.37 | 12.29144889 |
| MockStigmaLib4 | 7840358 | 79.24 | 8.711508889 |
| MockTTLib1 | 14926364 | 84.52 | 16.58484889 |
| MockTTLib3 | 3630257 | 73.83 | 4.033618889 |
| MockTTLib4 | 1942409 | 83.12 | 2.158232222 |
| MockBaseLib1 | 8996380 | 83.46 | 9.995977778 |
| MockBaseLib3 | 13532668 | 84.26 | 15.03629778 |
| MockBaseLib4 | 10663021 | 84.14 | 11.84780111 |
| CpStigmaLib2 | 14346498 | 79.38 | 15.94055333 |
| CpStigmaLib5 | 2016424 | 78.79 | 2.240471111 |
| CpTTLib1 | 9443334 | 83.24 | 10.49259333 |
| CpTTLib2 | 6285660 | 83.19 | 6.984066667 |
| CpTTLib5 | 2906021 | 82.51 | 3.228912222 |
| CpBaseLib1 | 20206113 | 76.82 | 22.45123667 |
| CpBaseLib2 | 12578496 | 82.87 | 13.97610667 |
| CpBaseLib5 | 6087878 | 83.95 | 6.764308889 |
| **48 hours** |  |  |  |
| MockTTLib2 | 7569385 | 84.89 | 8.410427778 |
| MockTTLib4 | 11137831 | 83.71 | 12.37536778 |
| MockTTLib5 | 4312611 | 83.64 | 4.79179 |
| MockBaseLib2 | 12703101 | 84.76 | 14.11455667 |
| MockBaseLib4 | 5248668 | 83.65 | 5.831853333 |
| MockBaseLib5 | 5189031 | 84.11 | 5.76559 |
| CpTTLib1 | 10173382 | 82.79 | 11.30375778 |
| CpTTLib2T | 4245053 | 80.79 | 4.716725556 |
| CpTTLib5 | 8949836 | 84.1 | 9.944262222 |
| CpBaseLib1 | 14567310 | 83.88 | 16.1859 |
| CpBaseLib2 | 10873847 | 83.26 | 12.08205222 |
| CpBaseLib5 | 7322356 | 83.59 | 8.135951111 |
| **72 hours** |  |  |  |
| MockTTLib1 | 8230101 | 83.24 | 9.144556667 |
| MockTTLib2 | 9334661 | 80.54 | 10.37184556 |
| MockTTLib4 | 11068654 | 82.04 | 12.29850444 |
| MockBaseLib1 | 2925436 | 84.73 | 3.250484444 |
| MockBaseLib2 | 9009199 | 83.58 | 10.01022111 |
| MockBaseLib3 | 3226946 | 83.89 | 3.585495556 |
| CpTTLib1 | 8182526 | 83.47 | 9.091695556 |
| CpTTLib2 | 5640401 | 83.6 | 6.267112222 |
| CpTTLib4 | 2599522 | 71.53 | 2.888357778 |
| CpBaseLib1 | 13473051 | 84.21 | 14.97005667 |
| CpBaseLib2 | 10309054 | 83.62 | 11.45450444 |
| CpBaseLib4 | 9270608 | 82.79 | 10.30067556 |
| **5 days** |  |  |  |
| MockTTLib1 | 4083856 | 81.48 | 4.537617778 |
| MockTTLib4 | 6065295 | 82.39 | 6.739216667 |
| MockTTLib5 | 489017 | 64.66 | 0.543352222 |
| MockBaseLib1 | 5143207 | 83.23 | 5.714674444 |
| MockBaseLib4 | 5273324 | 83.92 | 5.859248889 |
| CpTTLib1 | 6021216 | 51.34 | 6.69024 |
| CpTTLib2 | 7411083 | 52.28 | 8.234536667 |
| CpTTLib4 | 12101102 | 53.75 | 13.44566889 |
| CpBaseLib1 | 9874448 | 53.15 | 10.97160889 |
| CpBaseLib2 | 7576524 | 56.18 | 8.41836 |
| CpBaseLib4 | 10439653 | 55.51 | 11.59961444 |
| **7 days** |  |  |  |
| MockTTLib3 | 2935220 | 79.94 | 3.261355556 |
| MockTTLib5 | 2410978 | 81.92 | 2.678864444 |
| MockBaseLib1 | 8124653 | 84.67 | 9.027392222 |
| MockBaseLib3 | 5635053 | 84.01 | 6.26117 |
| MockBaseLib5 | 11842104 | 84.29 | 13.15789333 |
| CpTTLib1 | 8740565 | 46.11 | 9.711738889 |
| CpTTLib2 | 5550154 | 50.12 | 6.166837778 |
| CpTTLib3 | 5232927 | 39.19 | 5.814363333 |
| CpBaseLib1 | 11398753 | 43.64 | 12.66528111 |
| CpBaseLib2 | 16517028 | 52.71 | 18.35225333 |
| CpBaseLib3 | 9605947 | 36.06 | 10.67327444 |

TT represents the RNA libraries made from ovary transmitting tissue.

30. The International Wheat Genome Sequencing Consortium (IWGSC), et al. 2018. Science, 17 (361) eaar7191 DOI: 10.1126/science.aar7191. Available online at: <https://wheat-urgi.versailles.inra.fr/>


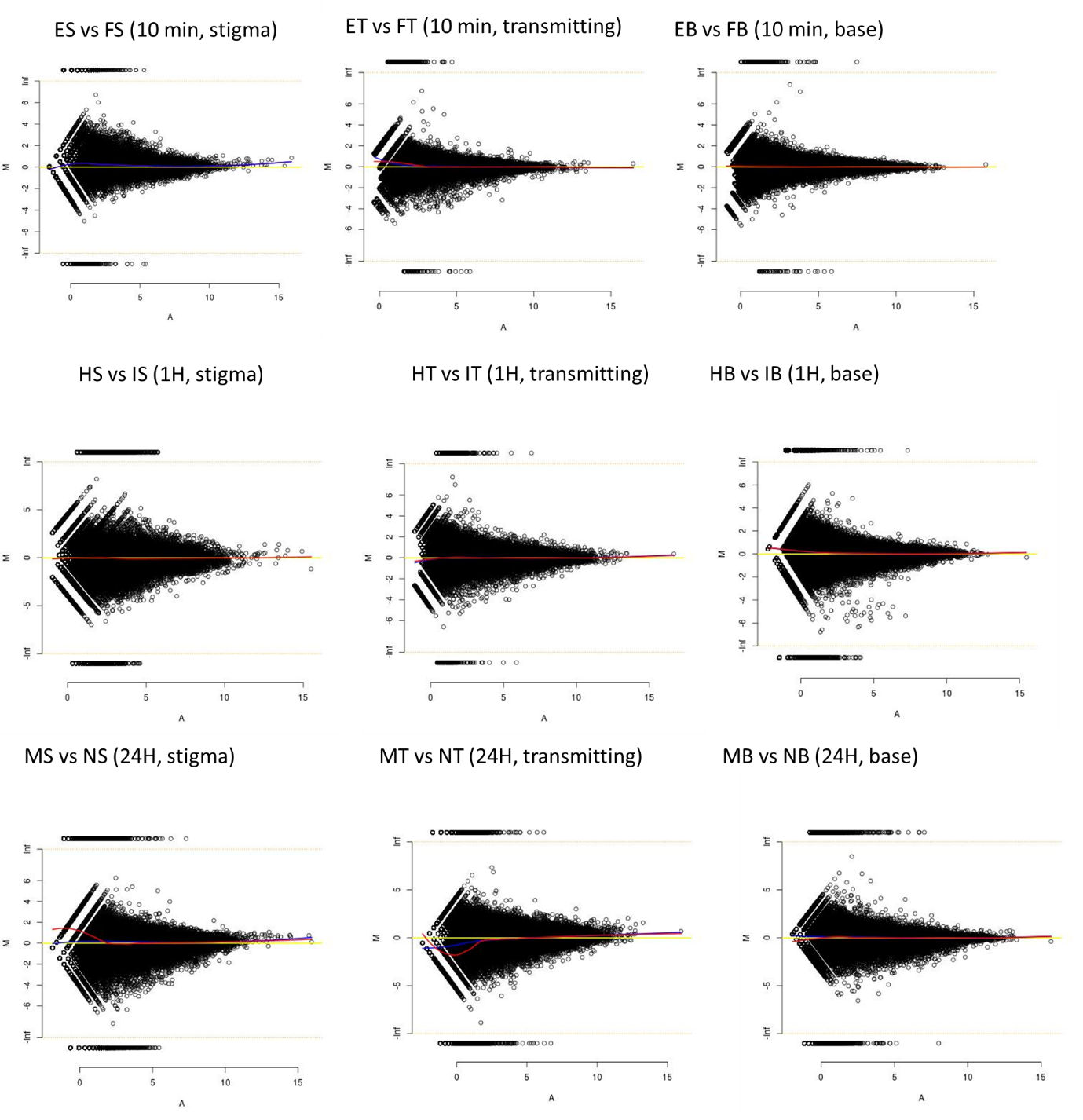


**Figure S1: MA plots for wheat transcripts at 10 mins, 1 hour and 24 hours.** Loess curves (red/blue) were drawn along with the line of symmetry at M=0 (yellow). The blue Loess curve are smoothened curves set at family = “symmetric”. The red is a regular Loess curve (M~A). In some figures, only one line is visible since two/all curves may overlap.


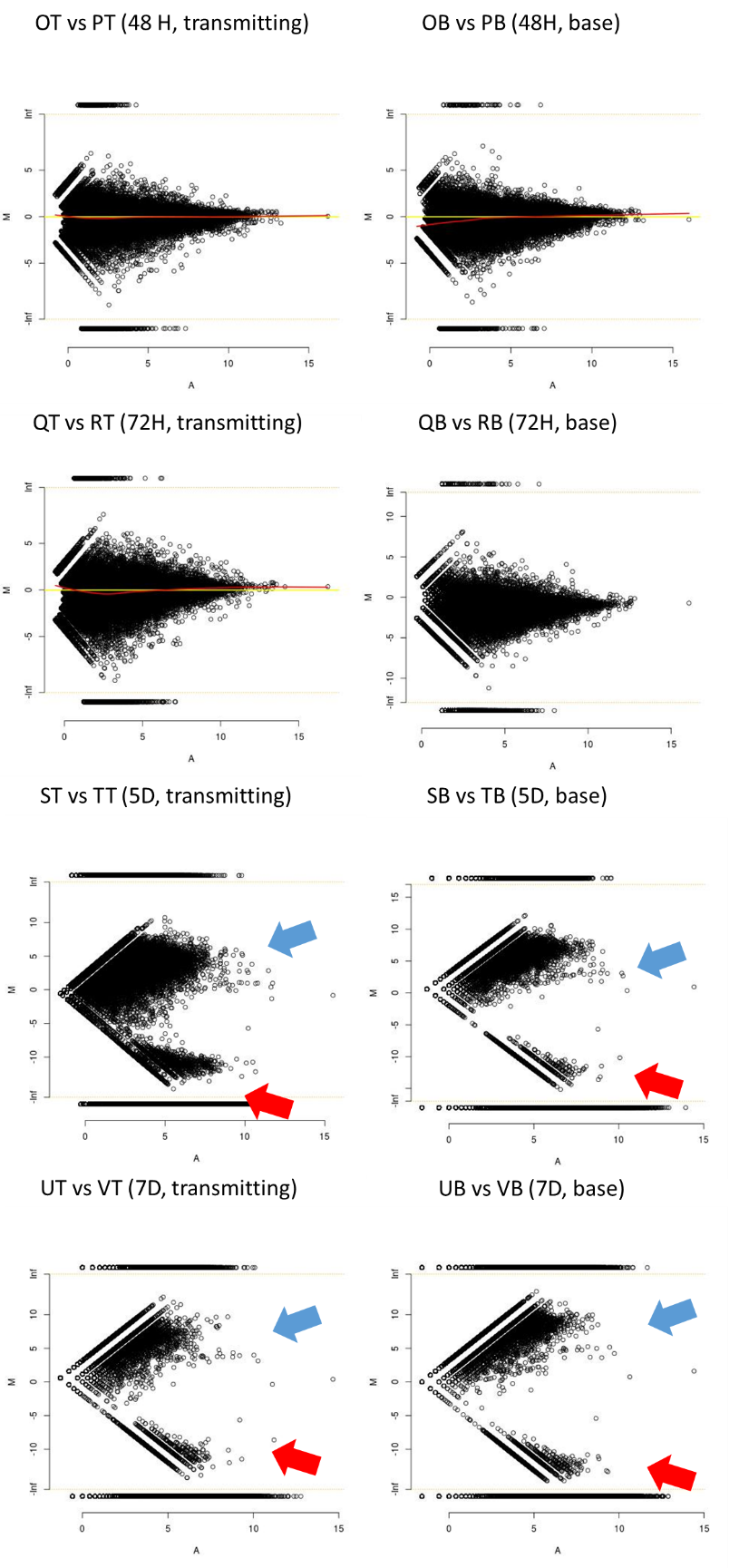


**Figure S2: MA plots for wheat transcripts at 48 hours, 72 hours, 5 days, and 7 days.** Loess curves (red/blue) were drawn along with the line of symmetry at M=0 (yellow). The blue Loess curve are smoothened curves set at family = “symmetric”. The red is a regular Loess curve (M~A). In some figures, only one line is visible since two/all curves may overlap. To demonstrate the asymmetric distribution of points, MA plots were generated using both wheat (blue arrow) and *C. purpurea* (red) transcripts.

**Table S2: Table of all differentially expressed hormone-related genes at the base tissue.** The red colour represents up-regulated genes and the green represents down-regulated genes. At the top, a schematic representation of the stage of fungal development in the wheat ovule at each time point is shown.

**
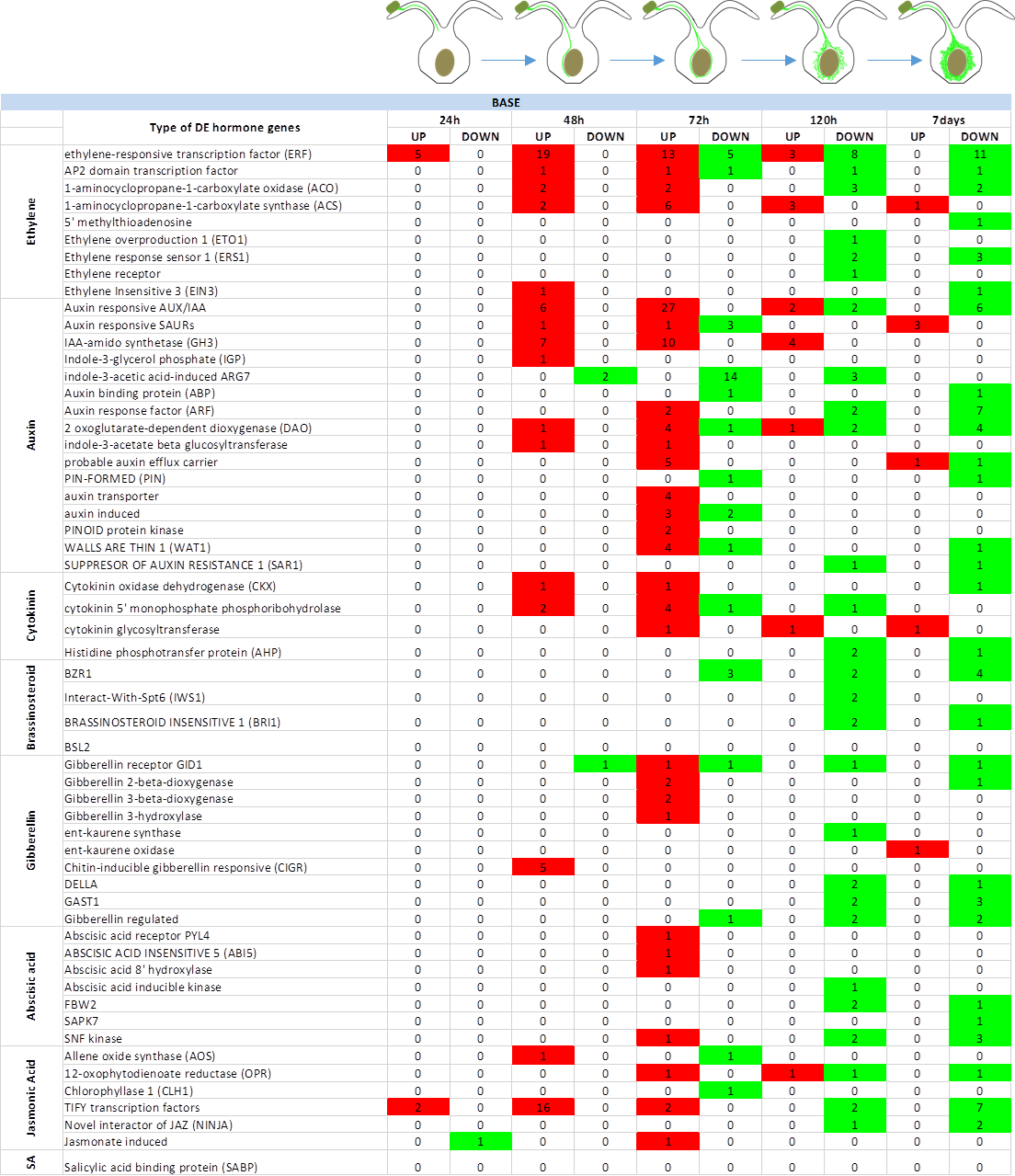
**

**Table S3: Table of all differentially expressed hormone-related genes at the transmitting tissue**. The red colour represents up-regulated genes and the green represents down-regulated genes. At the top, a schematic representation of the stage of fungal development in the wheat ovule at each time point is shown.

**
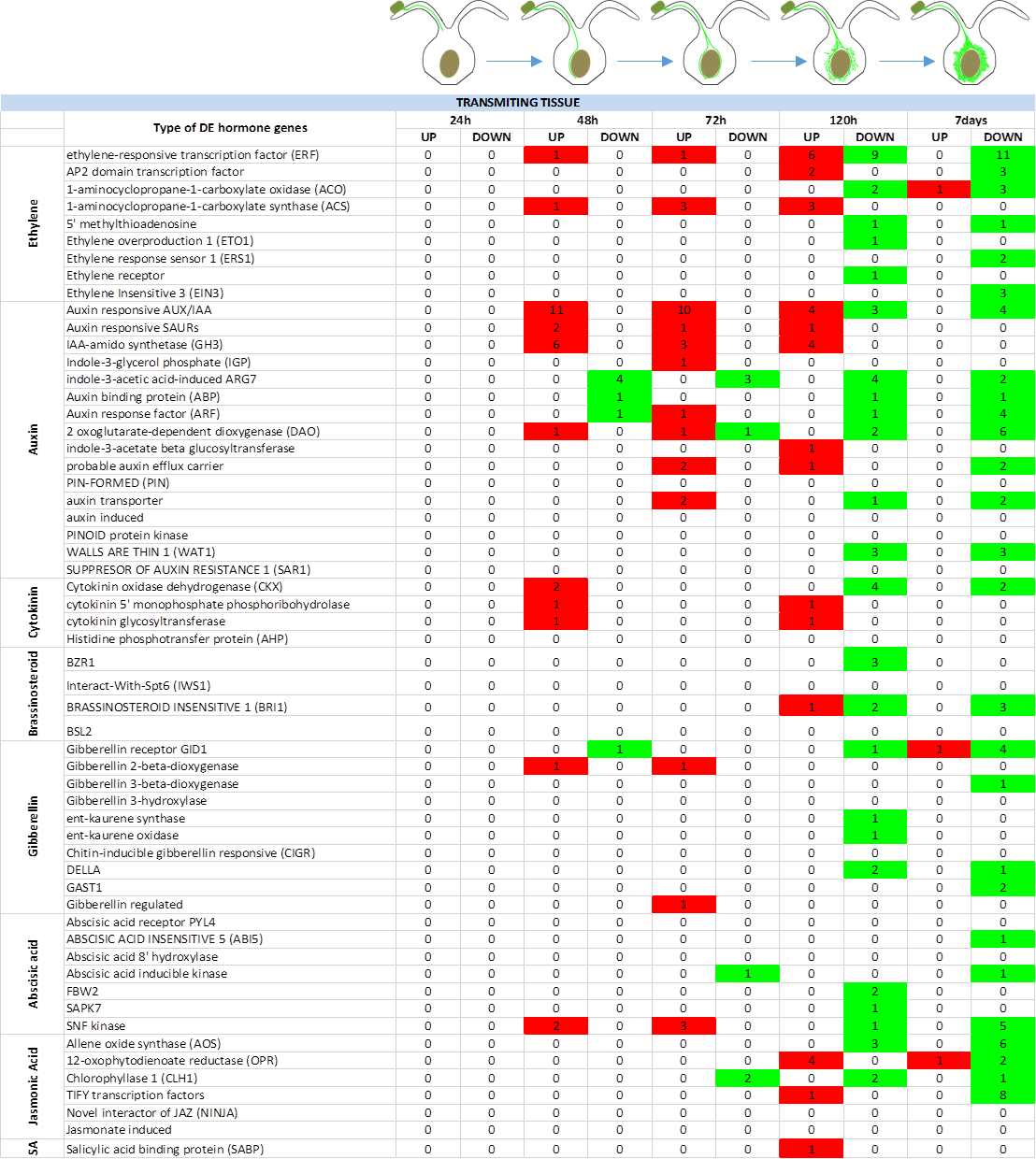
**

**Table S4: Table of all differentially expressed hormone-related genes at the stigma tissue.** The red colour represents up-regulated genes and the green represents down-regulated genes. At the top, a schematic representation of the stage of fungal development in the wheat ovule at each time point is shown.


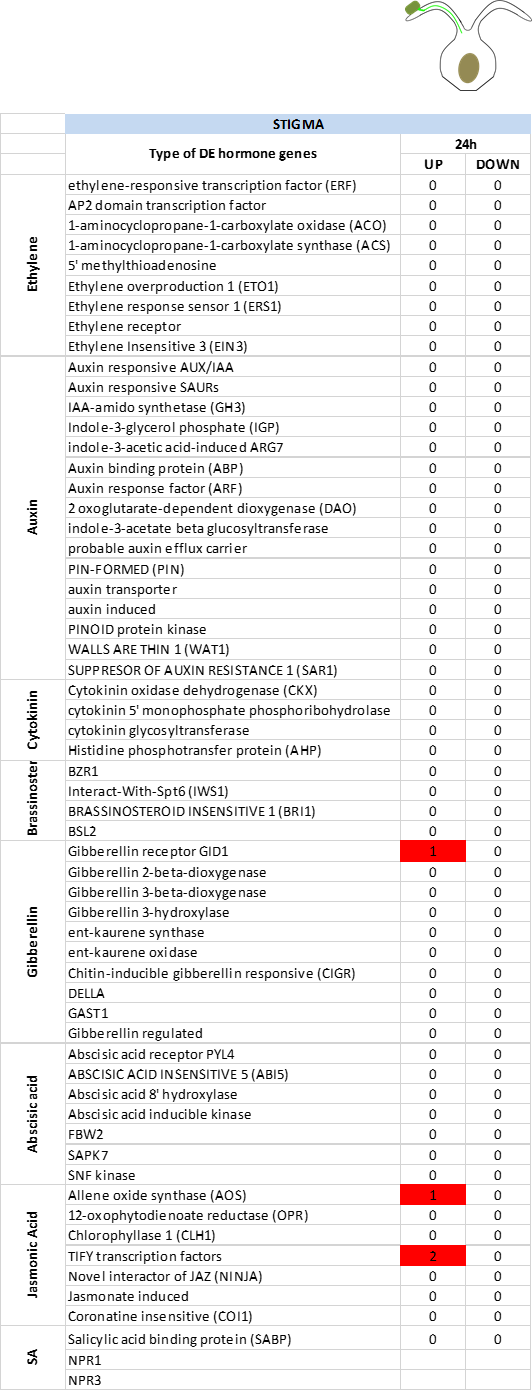


**Table S5: Table of all differentially expressed defence-related genes at the stigma tissue.** The red colour represents up-regulated genes and the green represents down-regulated genes. At the top, a schematic representation of the stage of fungal development in the wheat ovule at each time point is shown.


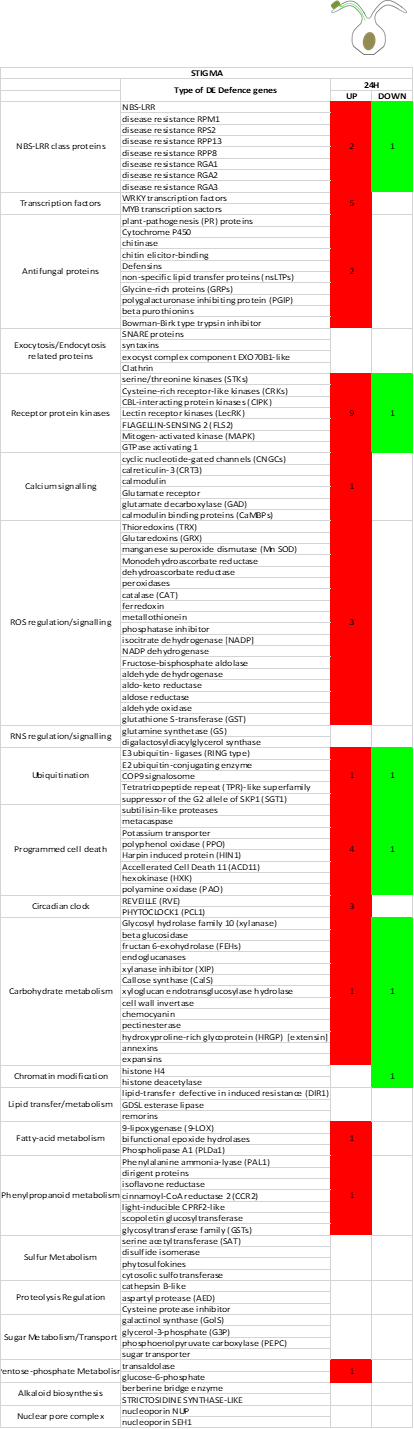


**Table S6: Table of all differentially expressed defence-related genes at the transmitting tissue.** The red colour represents up-regulated genes and the green represents down-regulated genes. At the top, a schematic representation of the stage of fungal development in the wheat ovule at each time point is shown.


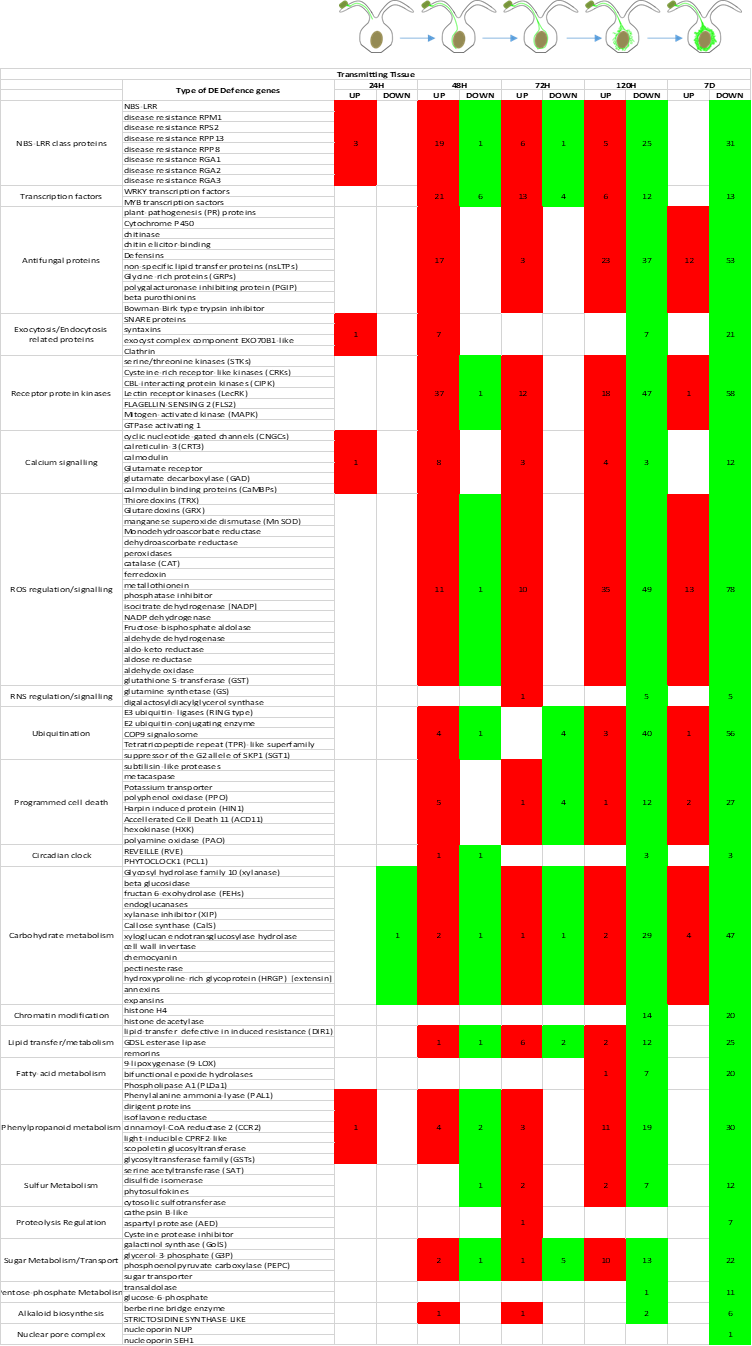


**Table S7: Table of all differentially expressed defence-related genes at the Base tissue.** The red colour represents up-regulated genes and the green represents down-regulated genes. At the top, a schematic representation of the stage of fungal development in the wheat ovule at each time point is shown.


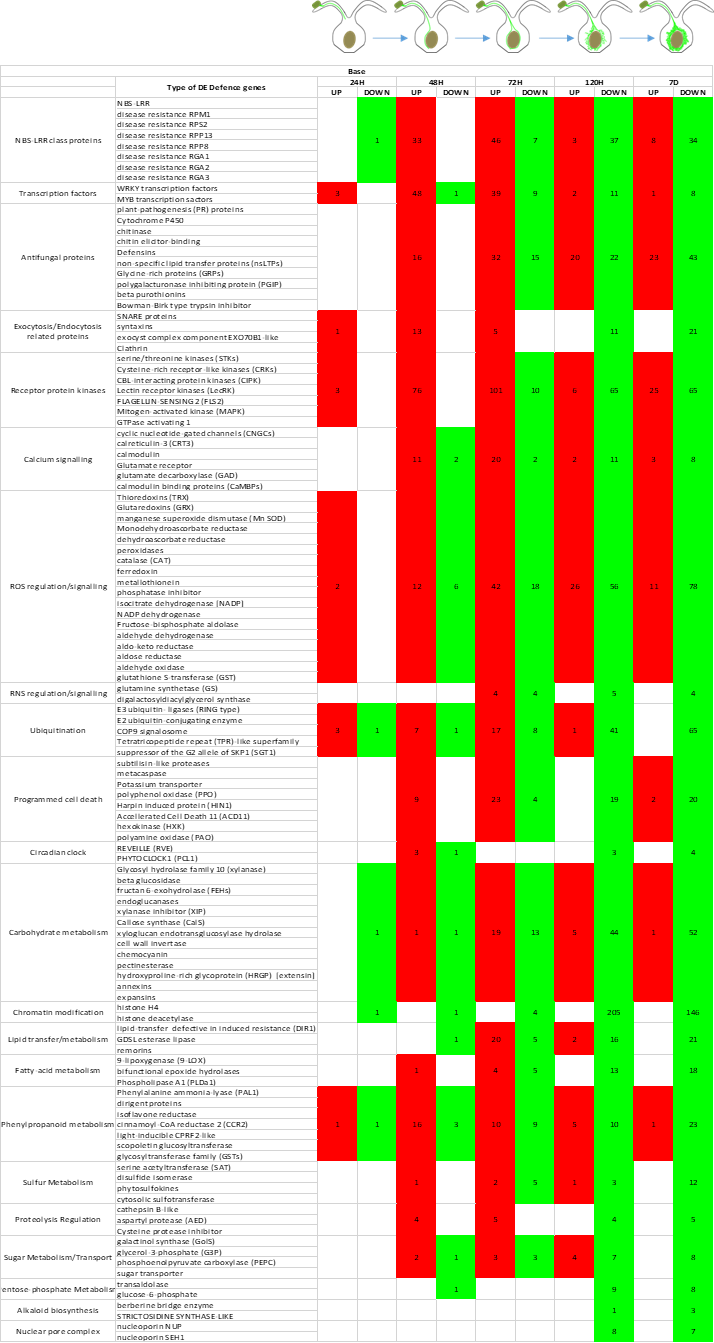

Supplement: Supplementary file 1 — Additional file 1: Fig. S1: MA plots for wheat transcripts at 10 mins, 1 h and 24 h. Loess curves (red/blue) were drawn along with the line of symmetry at M = 0 (yellow). The blue Loess curve are smoothened curves set at family = “symmetric”. The red is a regular Loess curve (M ~ A). In some figures, only one line is visible since two/all curves may overlap. Fig. S2: MA plots for wheat transcripts at 48 h, 72 h, 5 days, and 7 days. Loess curves (red/blue) were drawn along with the line of symmetry at M = 0 (yellow). The blue Loess curve are smoothened curves set at family = “symmetric”. The red is a regular Loess curve (M ~ A). In some figures, only one line is visible since two/all curves may overlap. To demonstrate the asymmetric distribution of points, MA plots were generated using both wheat (blue arrow) and C. purpurea (red) transcripts. Table S1: Percentage alignment rates of pair-end reads from 114 Mock and Cp-inoculated libraries against the International Wheat Genome Sequencing Consortium (IWGSC) wheat genomic reference from wheat variety Chinese Spring [30]. Table S2: Table of all hormone-associated genes differentially expressed in base tissue. Red represents up-regulated genes and green down-regulated genes. A schematic representation of the stages of Claviceps purpurea development in the wheat ovary at each time point is shown at the top of the table. Table S3: Table of all hormone-associated genes differentially expressed in transmitting tissue. Red represents up-regulated genes and green down-regulated genes. A schematic representation of the stages of Claviceps purpurea development in the wheat ovary at each time point is shown at the top of the table. Table S4: Table of all hormone-associated genes differentially expressed in stigma tissue. Red represents up-regulated genes and green down-regulated genes. A schematic representation of the stages of Claviceps purpurea development in the wheat ovary at each time point is shown at the top of the table. [file 12870_2021_3086_MOESM1_ESM.docx]
