# Supplementary figures and images for: Reprogramming of the wheat transcriptome in response to infection with Claviceps purpurea, the causal agent of ergot
Source: BMC Plant Biol. 2021 Jul 2;21:316. doi: 10.1186/s12870-021-03086-3 (PMC8252325; doi:10.1186/s12870-021-03086-3)

## Slide 1
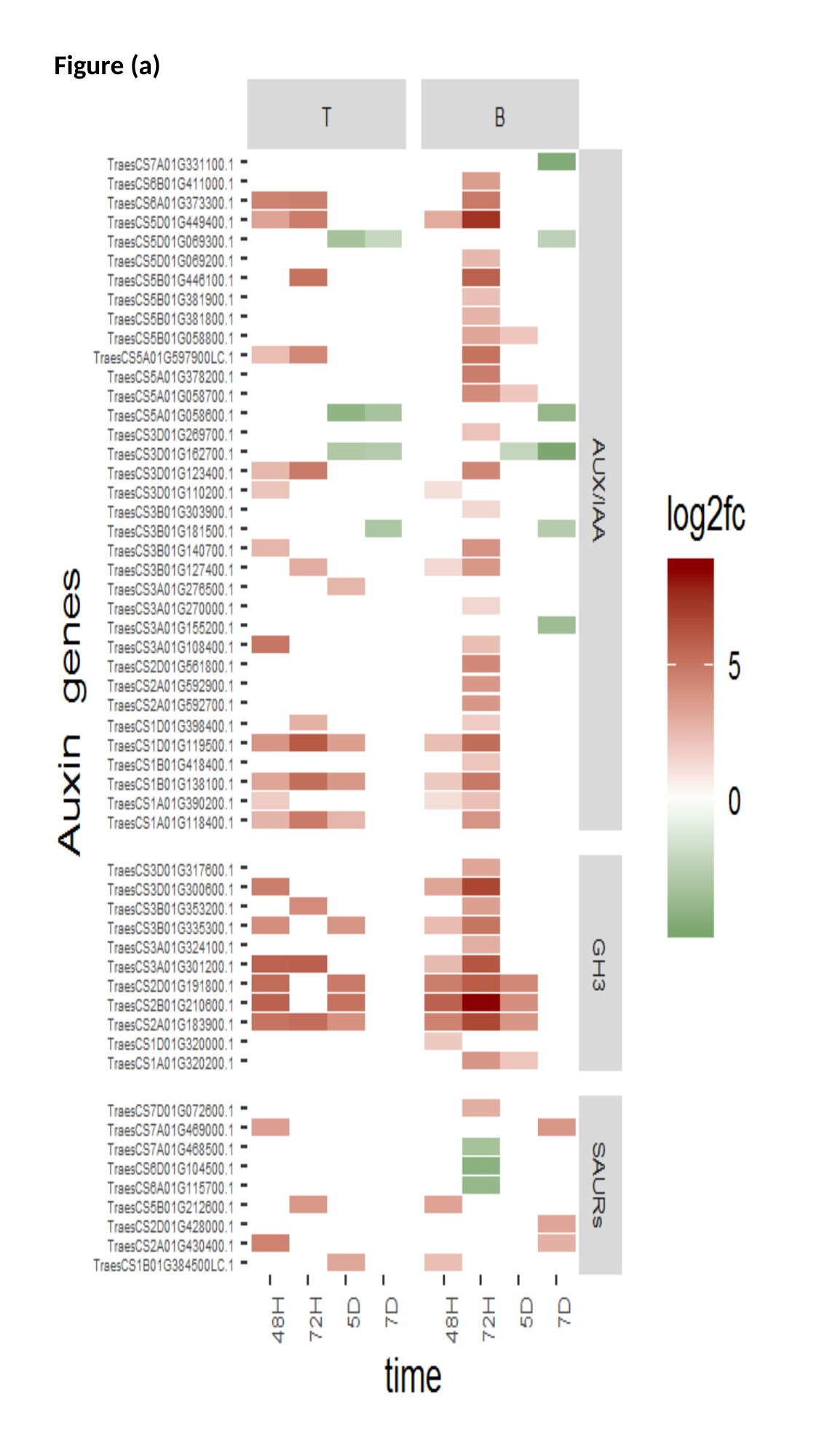

Figure (a)

## Slide 2
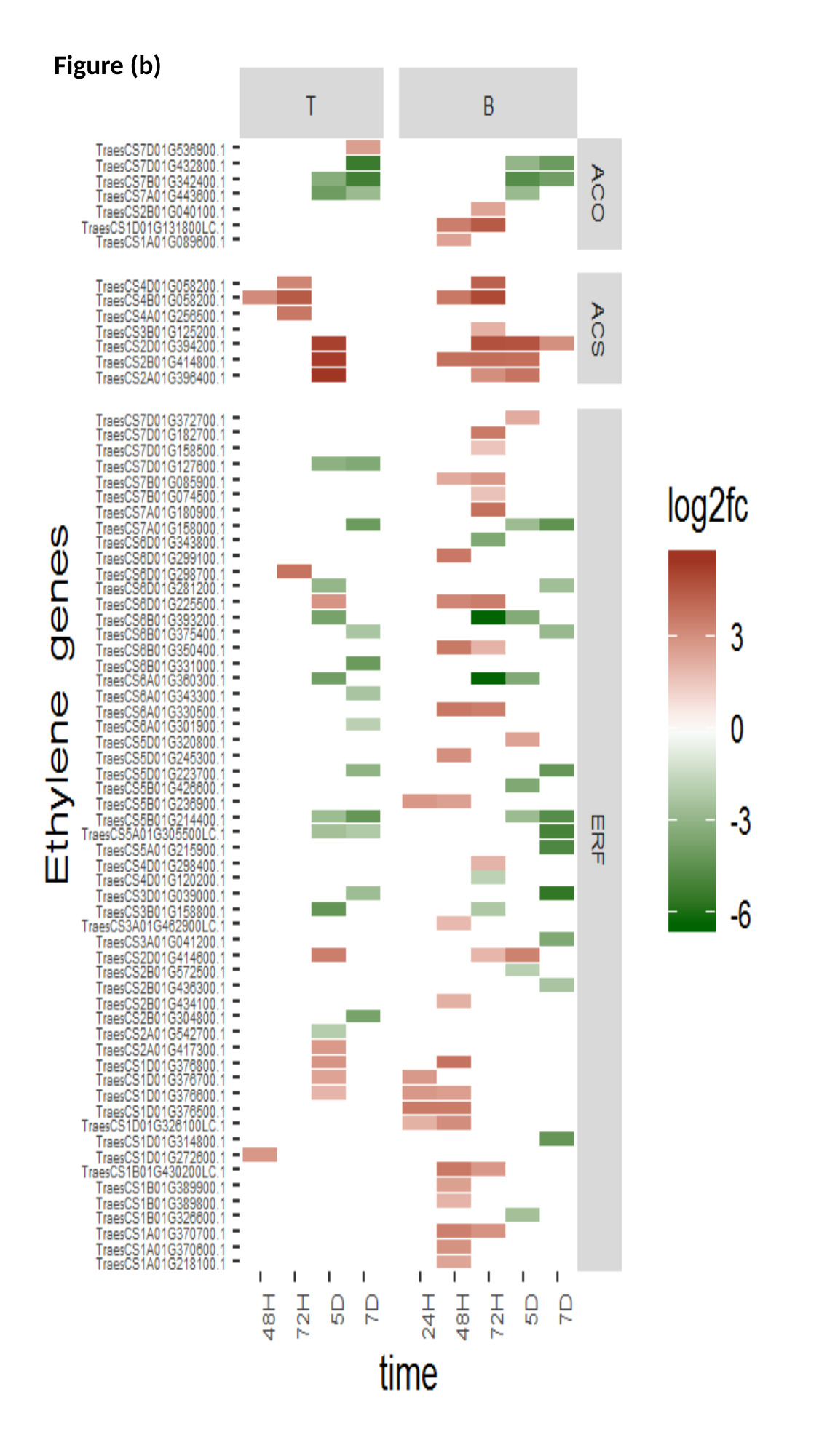

Figure (b)

## Slide 3
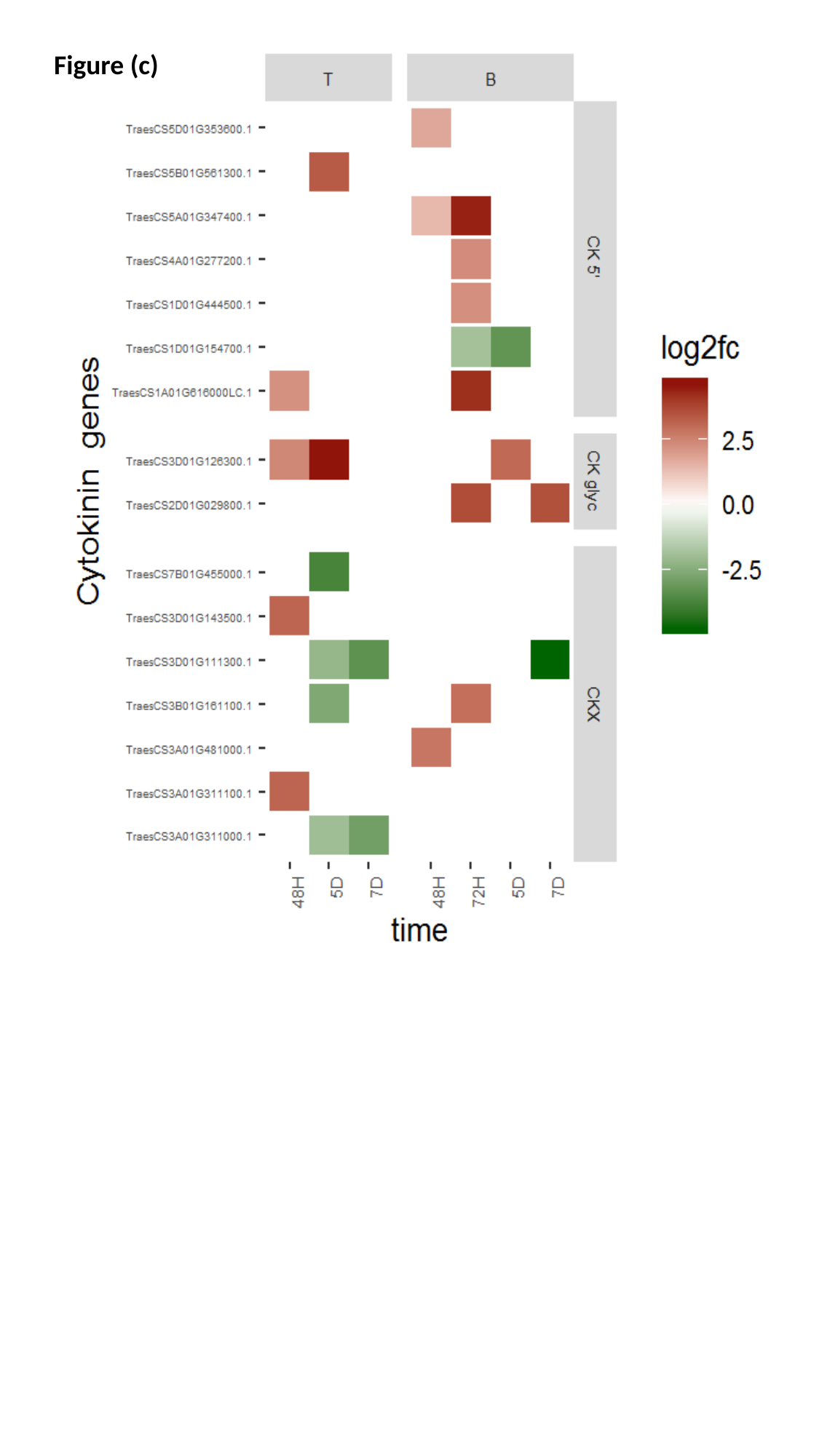

Figure (c)

## Slide 4
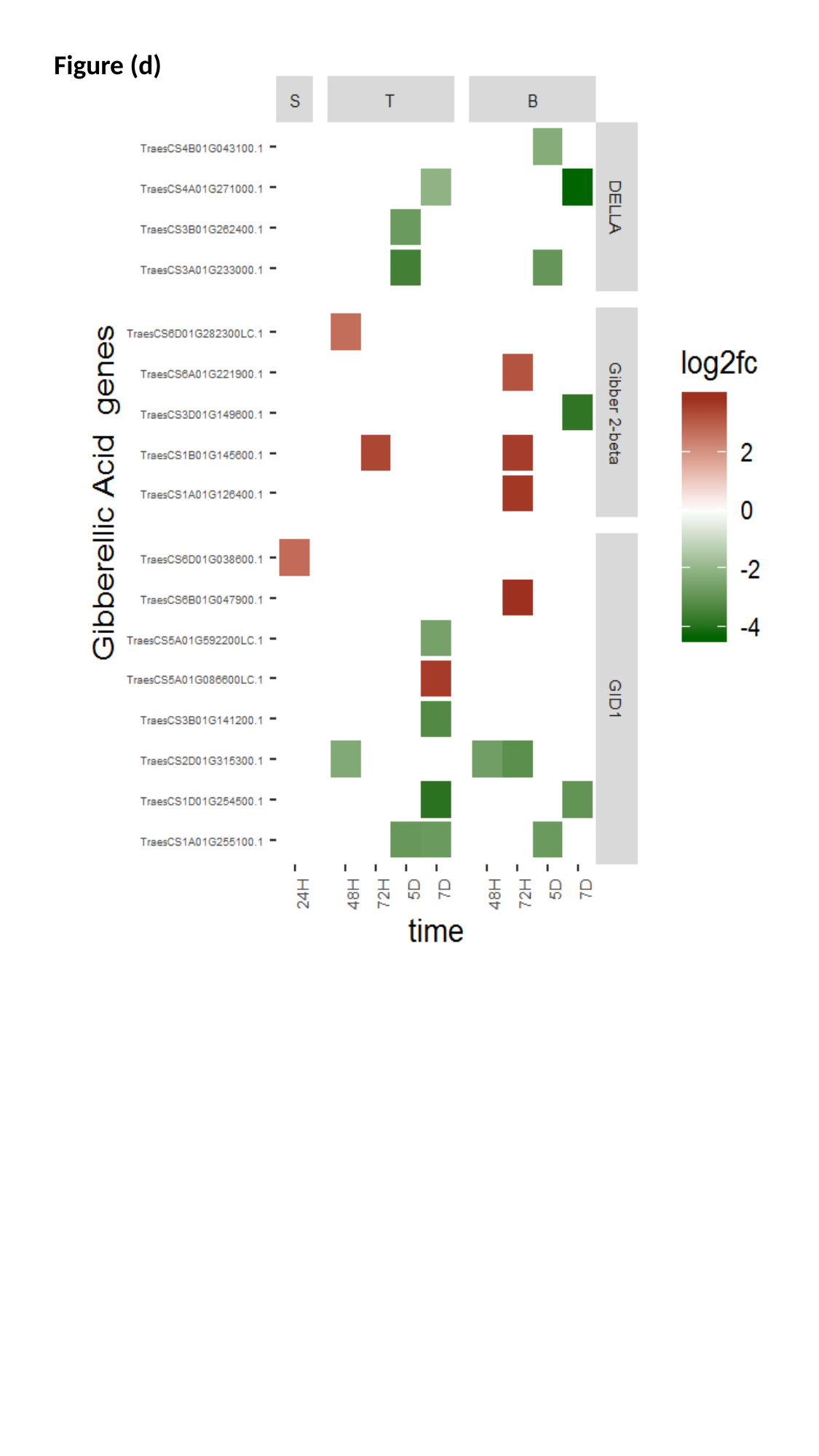

Figure (d)

## Slide 5
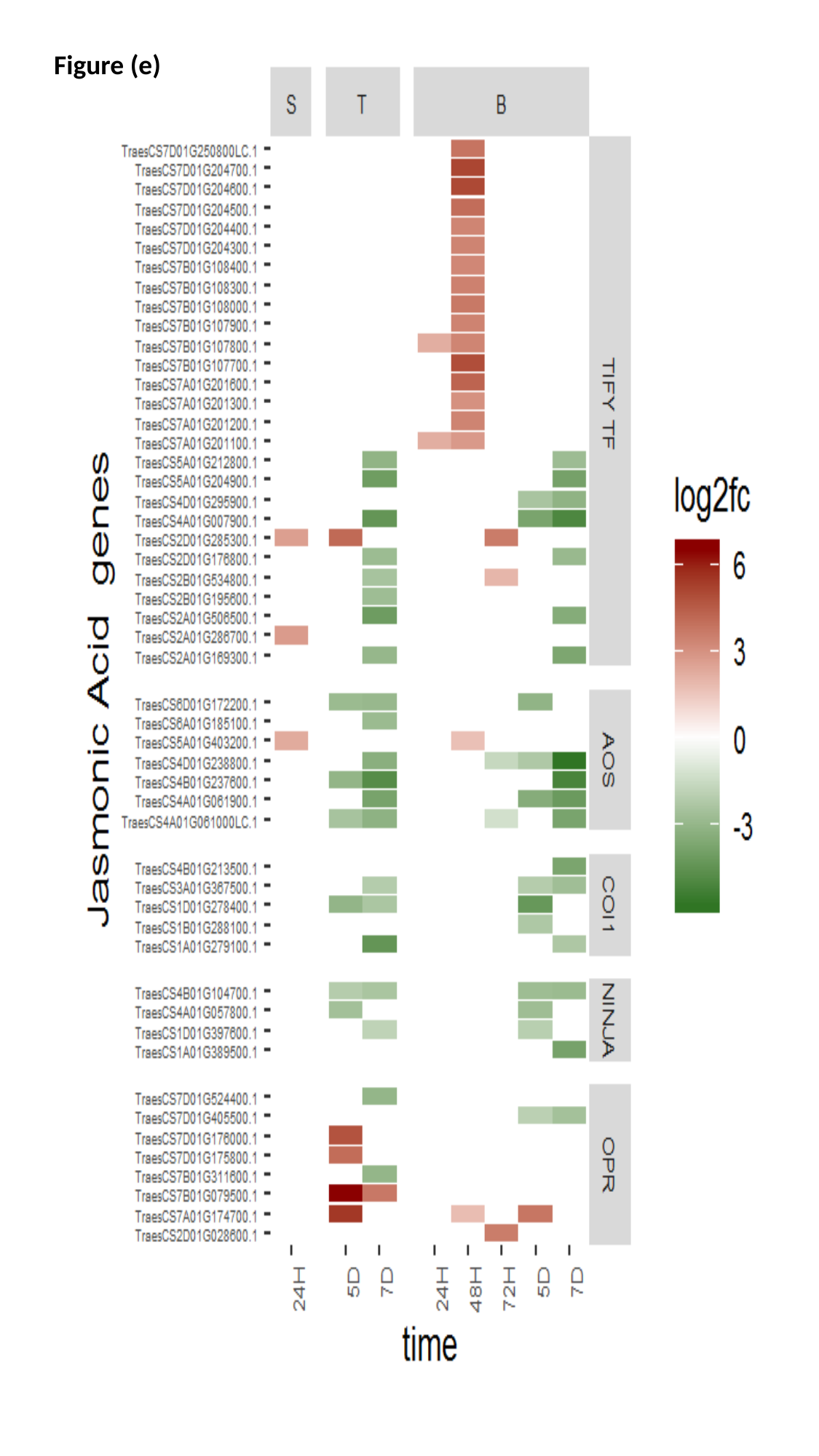

Figure (e)

## Slide 6
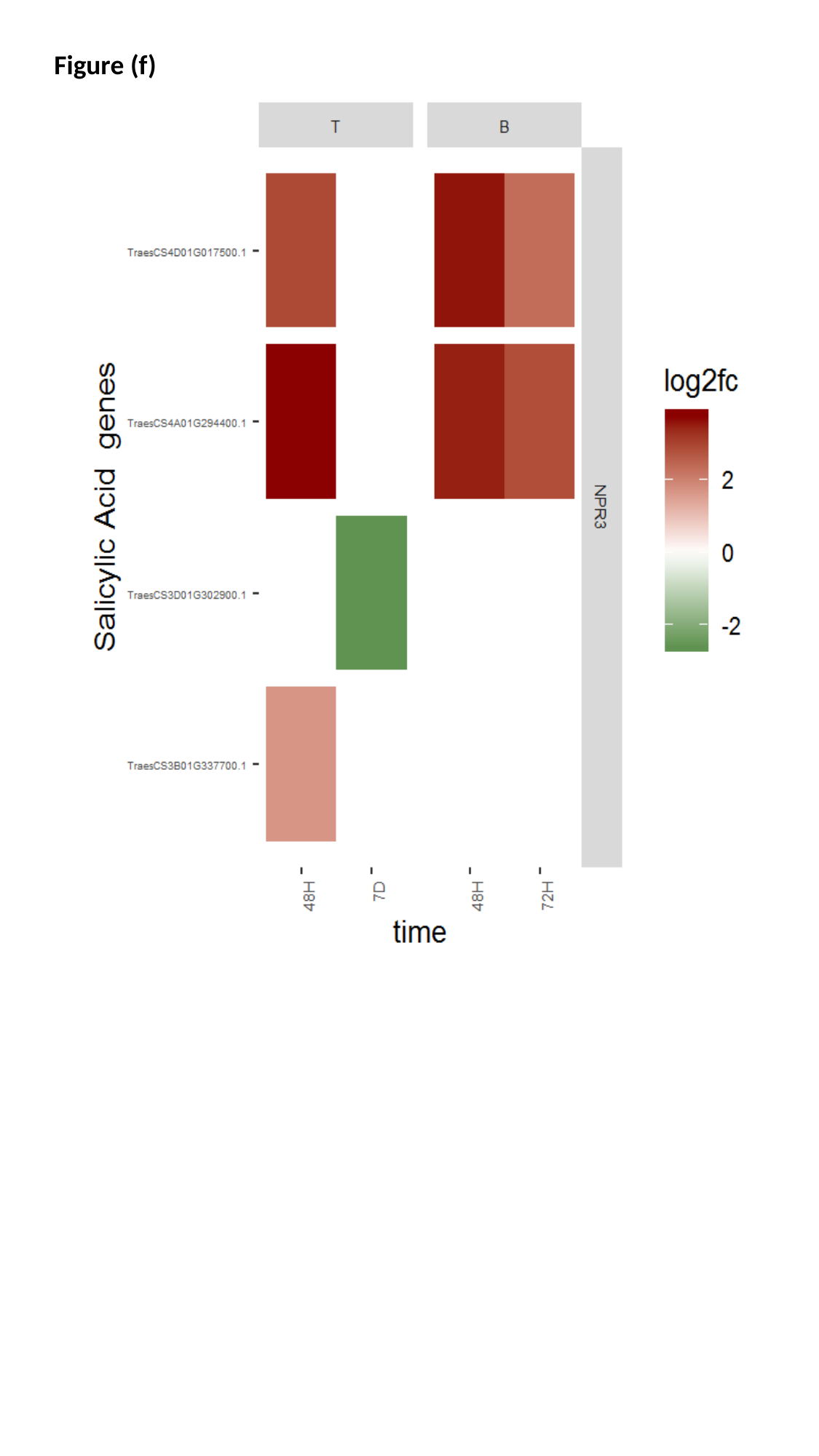

Figure (f)

Supplement: Supplementary file 5 — Additional file 5 Heatmaps of hormone-associated differentially expressed genes (DEG) across time points and tissues. DEG are defined by functional categories: Each figure shows the hormone-associated genes differentially up-regulated (log2 fold change in red) or down-regulated (in green) in wheat inoculated with Claviceps purpurea, relative to Mock-inoculated wheat, in stigma (S), transmitting (T) and base (B) tissues of the wheat ovary, at timepoints after inoculation: 24 h (24H), 48 h (48H), 72 h (72H), 5 days (5D) and 7 days (7D). Figure (a) Auxin-related genes ((Categories from top to bottom: Auxin/indole-3-acetic acid (AUX/IAA), Glycoside Hydrolase 3 (GH3), small Auxin-Up RNAs (SAURs)). Figure (b) Ethylene-related genes ((Categories from top to bottom: 1-Aminocyclopropane-1-carboxylate oxidase (ACO), 1-Aminocyclopropane-1-carboxylate synthase (ACS), Ethylene responsive transcription factors (ERF)). Figure (c) Cytokinin-related genes ((Categories from top to bottom: cytokinin riboside 5′-monophosphate phosphoribohydrolase (CK 5′), cytokinin specific glycosyltransferases (CK glyc), cytokinin oxidase/dehydrogenase (CKX)). Figure (d) Gibberellic acid-related genes ((Categories from top to bottom: DELLA, gibberellin 2-beta-oxidase (Gibber 2-beta), GA-INSENSITIVE DWARF1 (GID1)). Figure (e) Jasmonic acid-related genes ((Categories from top to bottom: TIFY transcription factors (TIFY TF), allene oxide synthase (AOS), coronatine-insensitive 1 (COI1), Novel INteractor of JAZ (NINJA), 12-oxophytodienoate reductase (OPR)). Figure (f) Salicylic acid-related genes ((Categories: NON-EXPRESSOR OF PR3 (NPR3)). Traes number refers to the gene annotation provided by the International Wheat Genome Sequencing Consortium (IWGSC) wheat genomic reference from wheat variety Chinese Spring [30].(PPTX 7966 kb) [file 12870_2021_3086_MOESM5_ESM.pptx]

## Slide 1
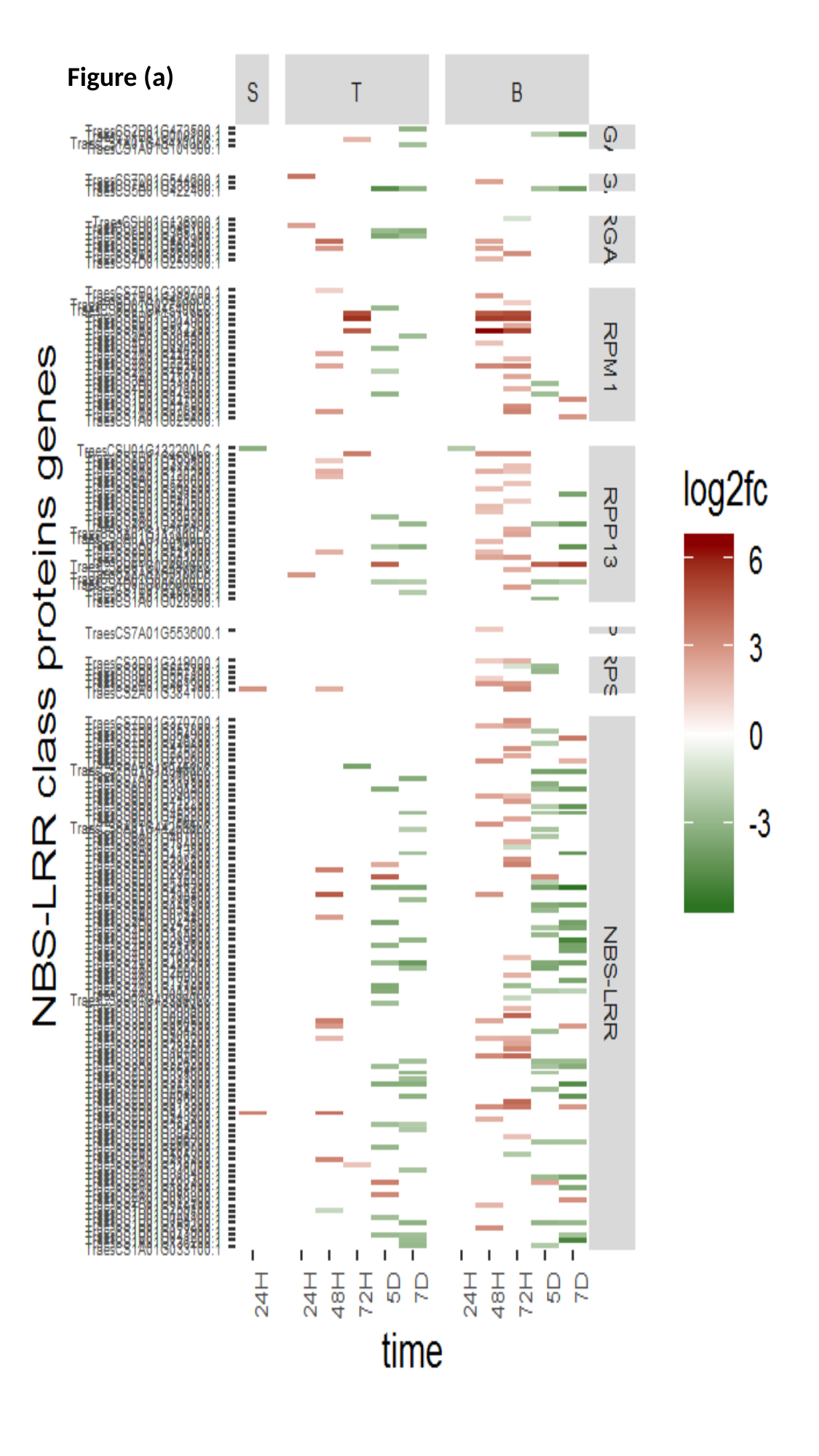

Figure (a)

## Slide 2
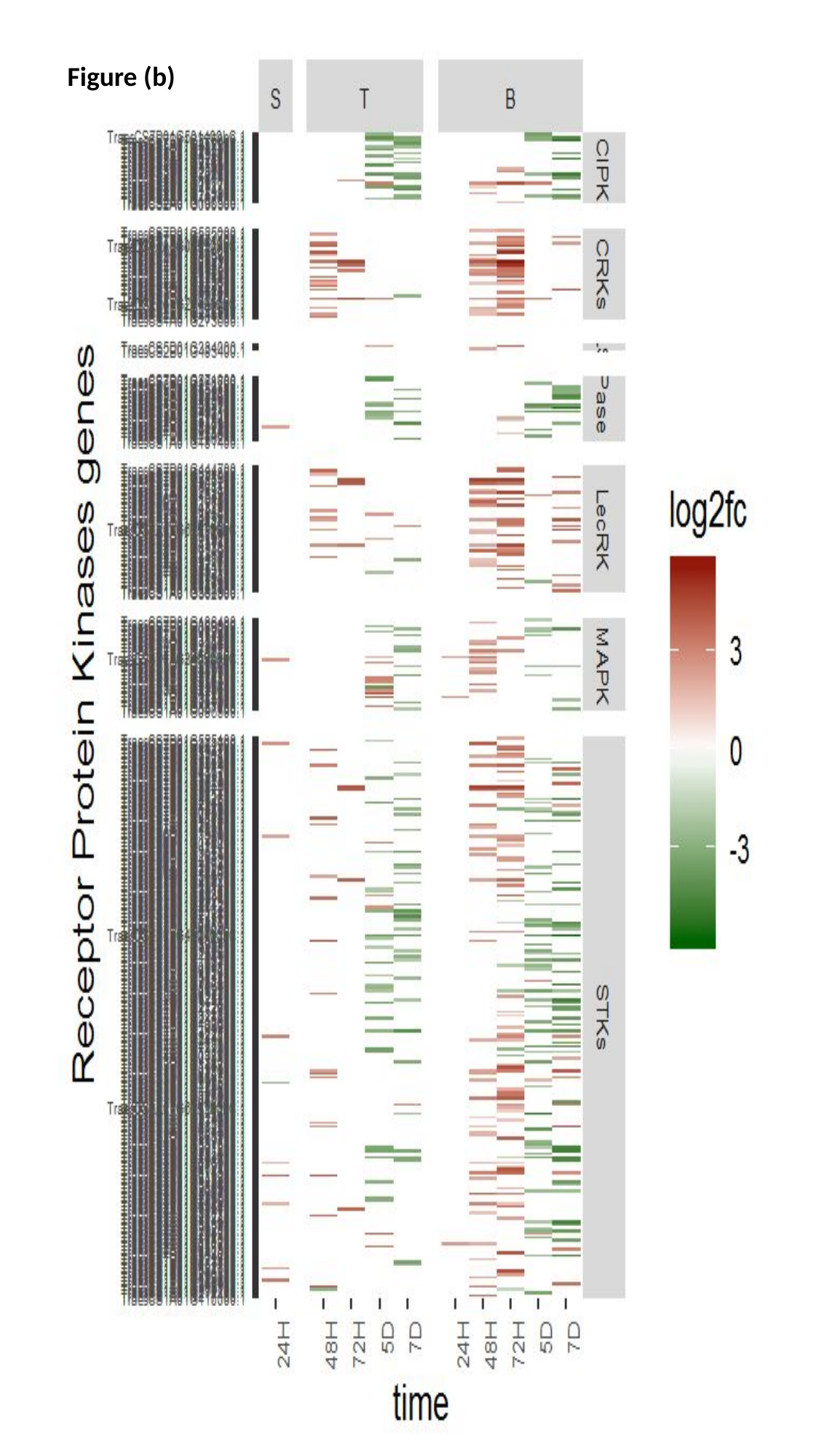

Figure (b)

## Slide 3
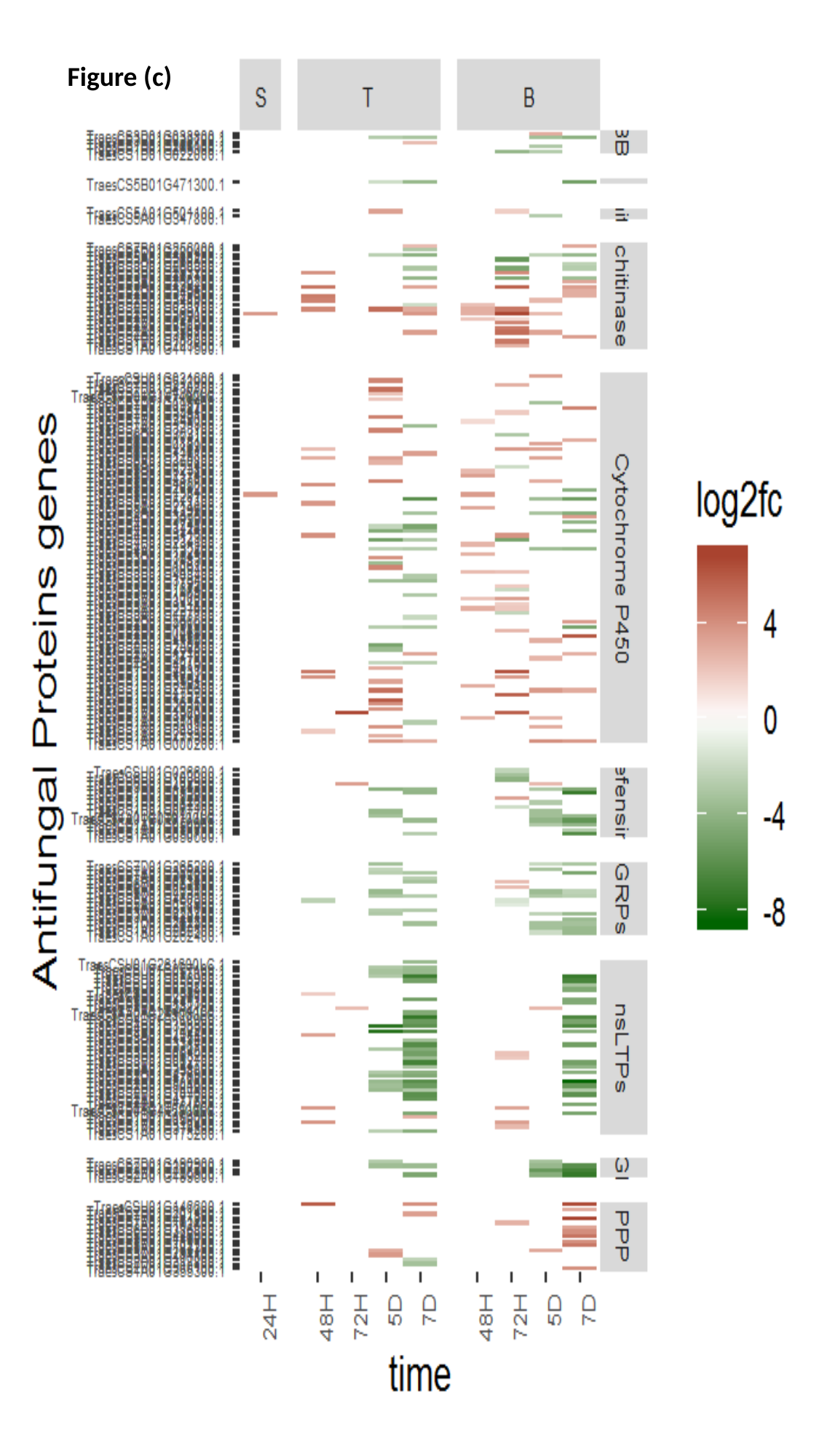

Figure (c)

## Slide 4
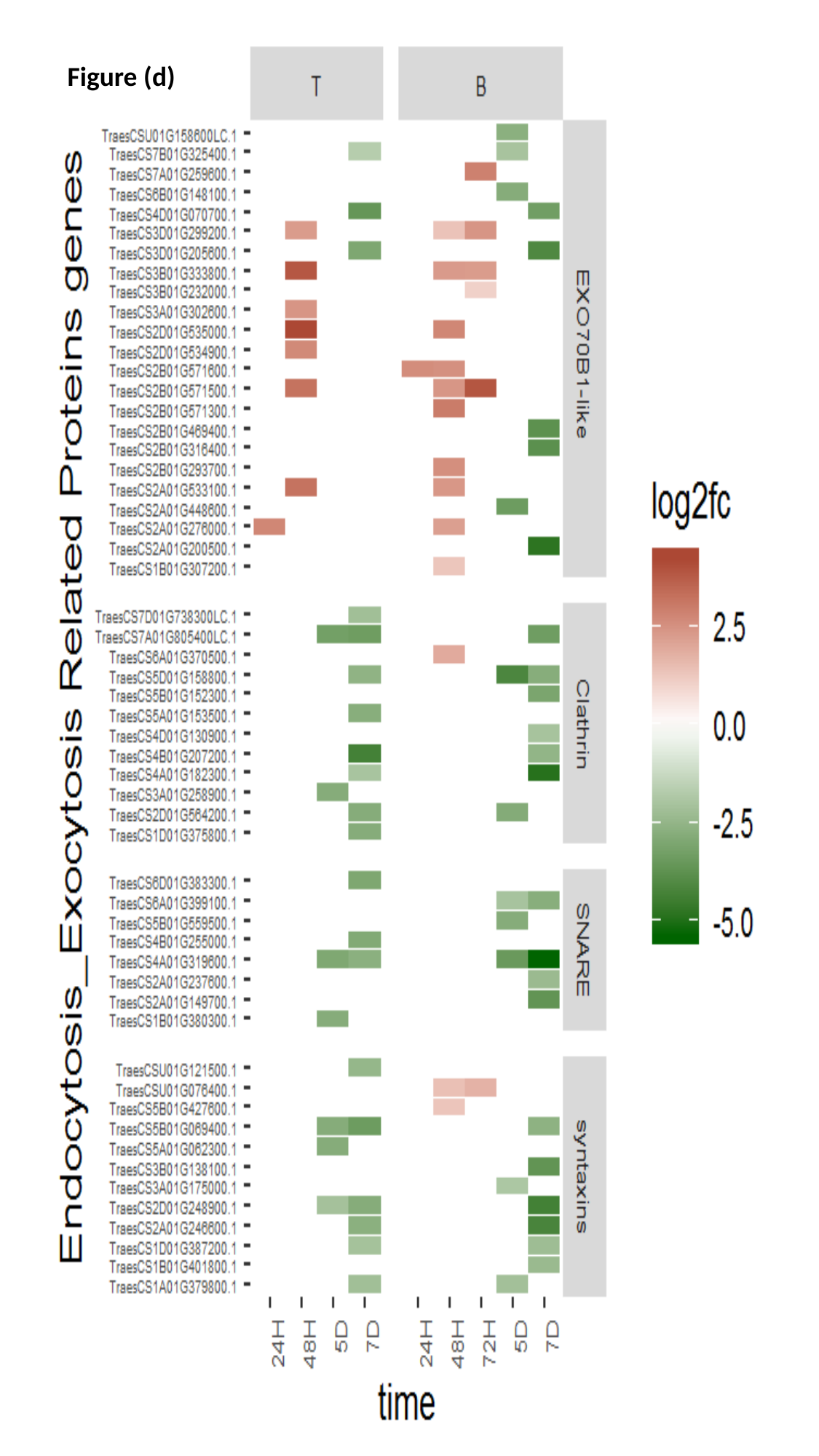

Figure (d)

## Slide 5
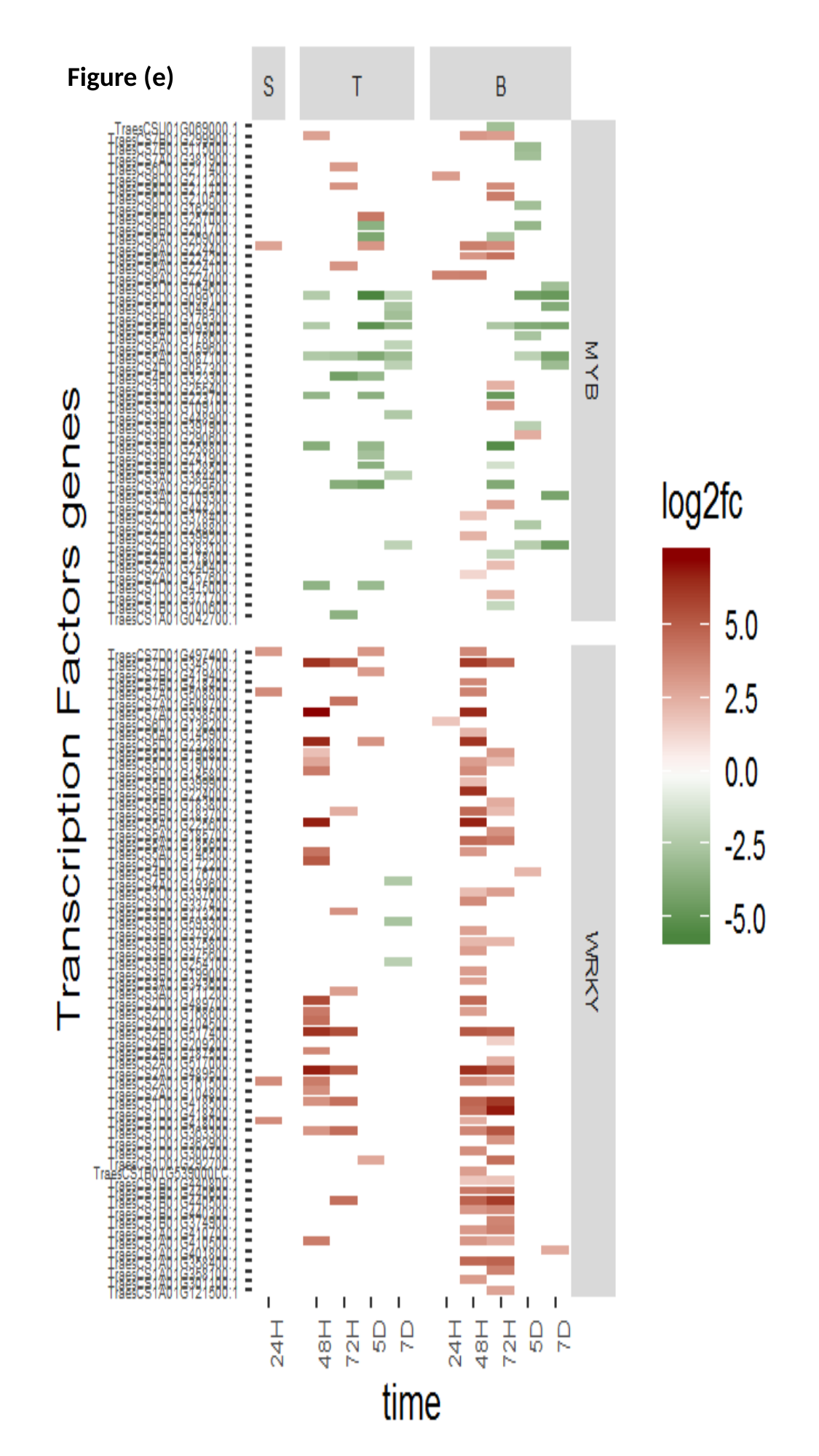

Figure (e)

## Slide 6
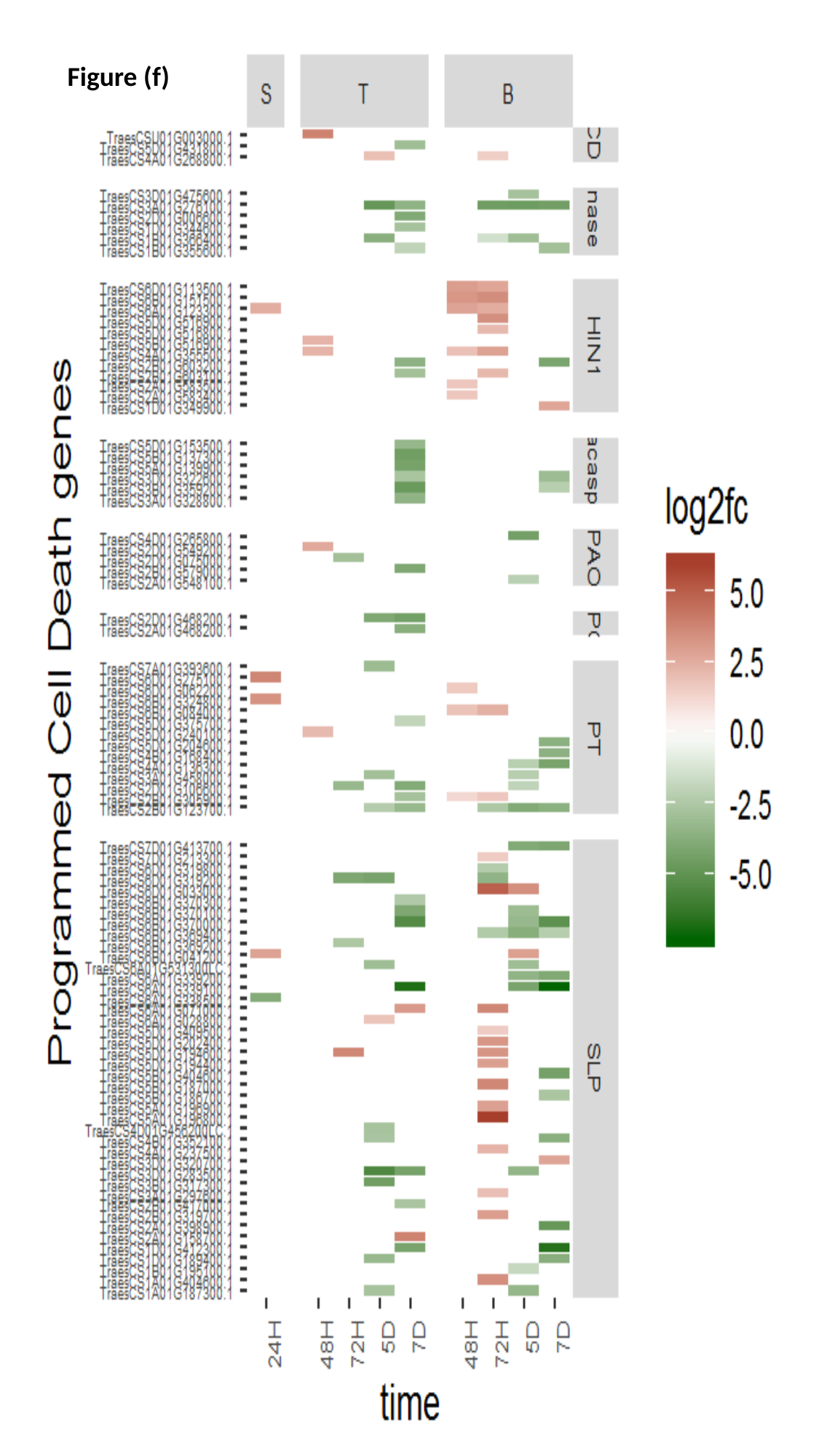

Figure (f)

Supplement: Supplementary file 6 — Additional file 6 Heatmaps of defence-related differentially expressed genes (DEG) across time points and tissues. DEG are defined by functional categories: Each figure shows the defence-related genes differentially up-regulated (log2 fold change in red) or down-regulated (in green) in wheat inoculated with Claviceps purpurea, relative to Mock-inoculated wheat, in stigma (S), transmitting (T) and base (B) tissues of the wheat ovary, at timepoints after inoculation: 24 h (24H), 48 h (48H), 72 h (72H), 5 days (5D) and 7 days (7D). Figure (a) NBS-LRR class proteins (functional categories from top to bottom: RGA1, RGA2, RGA3, RPM1, RPP13, RPP8, RPS2, NBS-LRR). Figure (b) Receptor protein kinases ()functional categories from top to bottom: CBL-interacting protein kinases (CIPK), Cysteine-rich receptor-like kinases (CRKs), Flagellin-sensing 2 (FLS2), GTPase activating 1, Lectin receptor kinases (LecRK), Mitogen-activated kinase (MAPK), serine/threonine kinases (STKs)). Figure (c) Antifungal proteins ((functional categories from top to bottom: Bowman-Birk type trypsin inhibitor (BBI), beta purothionins, chitin elicitor-binding, chitinase, Cytochrome P450, Defensins, Glycine-rich proteins (GRPs), non-specific lipid transfer proteins (nsLTPs), polygalacturonase inhibiting protein (PGIP), plant-pathogenesis proteins (PPP)). Figure (d) Endocytosis/Exocytosis related proteins. Figure (e) Transcription factors. Figure (f) Programmed cell death related genes ((functional categories from top to bottom: Accelerated Cell Death 11 (ACD11), hexokinase (HXK), Harpin induced protein (HIN1), metacaspase, polyamine oxidase (PAO), polyphenol oxidase (PPO), Potassium transporter (PT), subtilisin-like proteases (SLP)). Traes number refers to the gene annotation provided by the International Wheat Genome Sequencing Consortium (IWGSC) wheat genomic reference from wheat variety Chinese Spring [30].(PPTX 9371 kb) [file 12870_2021_3086_MOESM6_ESM.pptx]
